# QMR^®^ and Patient Blood-Derived Secretome Modulate RPE microRNA Networks Under Oxidative Stress

**DOI:** 10.3390/ijms26178614

**Published:** 2025-09-04

**Authors:** Simona Alibrandi, Domenico Mordà, Concetta Scimone, Angela D’ascola, Federica Aliquò, Alessandro Pozzato, Sergio Zaccaria Scalinci, Rosalia D’Angelo, Antonina Sidoti, Luigi Donato

**Affiliations:** 1 Department of Biomedical and Dental Sciences and Morphofunctional Imaging, Division of Medical Biotechnologies and Preventive Medicine, University of Messina, 98125 Messina, Italy; salibrandi@unime.it (S.A.); domenico.morda@studenti.unime.it (D.M.); cscimone@unime.it (C.S.); federica.aliquo@unime.it (F.A.); rdangelo@unime.it (R.D.); ldonato@unime.it (L.D.); 2 Department of Biomolecular Strategies, Genetics and Avant-Garde Therapies, Euro-Mediterranean Institute of Science and Technology (I.E.ME.S.T.), 90139 Palermo, Italy; 3 Department of Veterinary Sciences, University of Messina, 98125 Messina, Italy; 4 Department of Clinical and Experimental Medicine, University of Messina, 98125 Messina, Italy; angela.dascola@unime.it; 5 Telea Electronic Engineering Srl, 36066 Sandrigo, Italy; alessandro.pozzato@teleamedical.com; 6 Department of Medical and Surgical Sciences, University of Bologna, 40121 Bologna, Italy; sergio.scalinci@unibo.it

**Keywords:** Quantum Molecular Resonance, microRNA, oxidative stress, retinal pigment epithelium, mesenchymal stromal cell secretome, apoptosis, fibrosis, age-related macular degeneration

## Abstract

Oxidative stress destabilizes microRNA homeostasis in the retinal pigment epithelium (RPE), driving apoptosis and the epithelial-to-mesenchymal transition, which contribute to age-related macular degeneration. We investigated whether Quantum Molecular Resonance (QMR^®^) electrostimulation, alone or combined with Patient Blood-Derived (PBD) secretoma, can reprogram the RPE miRNome and mitigate stress-induced damage. Human ARPE-19 cells were exposed to tert-butyl-hydroperoxide and treated with QMR^®^, PBD secretome, or their combination. The deep sequencing of small RNAs at 24 h and 72 h, followed by differential expression and pathway enrichment analyses, delineated treatment-driven miRNA signatures. Oxidative stress deregulated > 50 miRNAs, enriching pro-apoptotic, fibrotic, and inflammatory pathways. QMR^®^ restored roughly 40% of these miRNAs and upregulated additional cytoprotective species such as miR-590-3p, a known regulator of the NF-κB and NLRP3 pathways according to validated target databases. While these observations suggest the potential involvement of inflammatory and stress-related cascades, functional assays will be required to directly confirm such effects. Secretome treatment preferentially increased anti-inflammatory miR-146a-5p and regenerative miR-204-5p while suppressing pro-fibrotic let-7f-5p. Combined QMR^®^ + secretome triggered the broadest miRNA response, normalizing over two-thirds of stress-altered miRNAs. These changes are predicted to influence antioxidant, anti-apoptotic, and anti-fibrotic pathways, although they did not translate into additional short-term cytoprotection compared with QMR^®^ alone. These data indicate that QMR^®^ and PBD secretome modulate complementary miRNA programs that converge on stress response networks. This broader molecular reprogramming may reflect regulatory complementarity, but functional validation is needed to determine whether it provides benefits beyond those observed with QMR^®^ alone. These findings offer molecular insights into potential non-invasive, cell-free strategies for retinal degeneration, although in vivo validation will be required before any clinical translation to Age-Related Macular Degeneration (AMD) therapy.

## 1. Introduction

Age-related macular degeneration (AMD) and related inherited retinal dystrophies are driven by chronic degeneration of the retinal pigment epithelium (RPE), a monolayer that sustains photoreceptor metabolism, phagocytosis, and ion homeostasis [1,2]. Even moderate oxidative insults overwhelm RPE anti-oxidant capacity, triggering the epithelial to mesenchymal transition (EMT), sterile inflammation, and apoptosis, which ultimately jeopardize visual function [3,4,5,6,7,8,9]. In vitro, exposure of human ARPE 19 cells to tert-butyl-hydroperoxide (tBHP) is widely adopted to emulate this oxidative milieu: tBHP rapidly depletes glutathione, activates p38/MAPK and NF-κB signaling, and provokes cell death within hours [10,11,12], faithfully reproducing the transcriptomic and functional hallmarks observed in AMD donor tissue.

MicroRNAs (miRNAs) act as master post-transcriptional regulators of stress adaptation by pairing with complementary sequences in target mRNAs, thereby tuning translation and stability. Several miRNAs—such as miR 141, miR 146a, and the miR 200 family—have been implicated in redox balance, mitochondrial quality control, and VEGF secretion in RPE cells [13,14]. Dysregulation of a handful of miRNAs (e.g., miR 34a and miR 210) is sufficient to exaggerate oxidative damage or promote EMT, underscoring their therapeutic potential. Yet, beyond a few well studied loci, the broader miRNA landscape that orchestrates RPE survival under oxidative challenge—and, crucially, how it can be remodeled by emerging therapies—remains poorly defined [15,16].

Quantum Molecular Resonance (QMR^®^) is a non-ionizing, low-potency technology that uses high-frequency waves in the range of 4–64 MHz. Initially developed for very-low-temperature surgical dissection, QMR^®^ has recently been repurposed for regenerative applications, improving tear film stability and ocular surface inflammation in prospective clinical studies [17,18]. At the cellular level, oscillatory electric fields can modulate membrane potential, calcium micro domains, and cytoskeletal dynamics, but virtually nothing is known about the intracellular signaling events engaged by QMR^®^ in neural or epithelial ocular tissues. To date, no study has examined whether QMR^®^ can reprogram miRNA networks in RPE cells exposed to oxidative stress, representing a significant knowledge gap.

Complementary to physical stimulation, trophic support can be provided by secretoma extracted from the platelet-rich plasma of AMD patients. Secretomes are enriched in antioxidant enzymes, pro-survival cytokines, and exosome-embedded miRNAs, which collectively mitigate ROS accumulation, dampen inflammation, and promote angiogenic regeneration in degenerating retina [19]. We hypothesized that coupling QMR^®^ with a PDB secretome would deliver synergistic, multi-level protection, with QMR^®^ supplying an endogenous electrophysiological trigger while paracrine factors sustain cytoprotection and tissue remodeling.

Here, we interrogate the combined and individual effects of QMR^®^ stimulation and PDB secretome on oxidatively stressed ARPE 19 cultures. Using deep small RNA sequencing across early (24 h) and late (72 h) phases after tBHP insult, we undertake the following:

1. Define the time-resolved miRNA signature induced by QMR^®^, PDB secretome, and their combination.
2. Integrate differential expression profiles with validated and in silico-predicted miRNA targetomes to reconstruct regulatory networks.
3. Identify the signaling pathways and biological processes most susceptible to QMR^®^-mediated modulation, thereby elucidating the mechanistic bases of the observed cytoprotection.

This work provides the first comprehensive view of how non-thermal radio frequency therapy reshapes post-transcriptional regulation in oxidatively challenged RPE, laying the molecular groundwork for its rational deployment—and biomarker monitoring—in ophthalmic therapeutics.

## 2. Results

### 2.1. MTT Cell Viability Assay Reveals QMR^®^-Mediated Cytoprotection Under Oxidative Stress

Exposure to tBHP for 24–72 h markedly reduced ARPE-19 cell viability, as reflected by the drop in MTT absorbance in the oxidative stress control (CTRL-OX) compared to untreated cells. At 24 h, the mean OD_570 for CTRL cells was 2696 (arbitrary units), whereas CTRL-OX cells dropped to 1376, roughly 50% of the control, confirming the cytotoxic impact of oxidative stress.

Treatment with QMR^®^ significantly rescued cell viability: the QMR^®^ group showed an absorbance of 2390 at 24 h, nearly restoring viability to the level of untreated controls. In contrast, cells receiving the secretome alone did not exhibit any appreciable protection; the SECRETOME group’s absorbance was only 0.892 ± SD (arbitrary OD 570 units at 570 nm, *n* = 3) at 24 h, indicating no improvement (and even a slight decline) in viability relative to the oxidized control. This lack of cytoprotection may reflect suboptimal concentration, variability in the bioactive factor composition of the patient-derived secretome, or the limited sensitivity of ARPE-19 cells to paracrine cues under acute oxidative stress. Notably, the combined treatment (RES + SECRETOME) yielded an absorbance of 1374 at 24 h—essentially identical to the CTRL-OX group—suggesting that adding the secretome provided no additive benefit over QMR^®^ alone. This suggests that the robust survival effect of QMR^®^ may saturate key protective pathways, while secretome-derived cues act on processes not directly captured by the MTT assay.

A similar pattern was observed at the 72 h time point. CTRL cells maintained a high viability (absorbance 1892), whereas tBHP exposure (CTRL-OX) reduced this to 1068. QMR^®^ again conferred robust cytoprotection: QMR^®^-treated cells had an OD_570 of 2198 at 72 h, exceeding that of the untreated control and indicating sustained cell survival and proliferation. However, secretome treatment alone remained ineffective (absorbance of 0.258 at 72 h, corresponding to a severely reduced viability), and the combination of RES + SECRETOME yielded an absorbance of 0.959—not outperforming QMR^®^ alone. In contrast, the secretome by itself provided minimal protection in this model, and co-treatment with QMR^®^ did not further improve viability beyond the effect of QMR^®^ alone.

**Figure 1:**
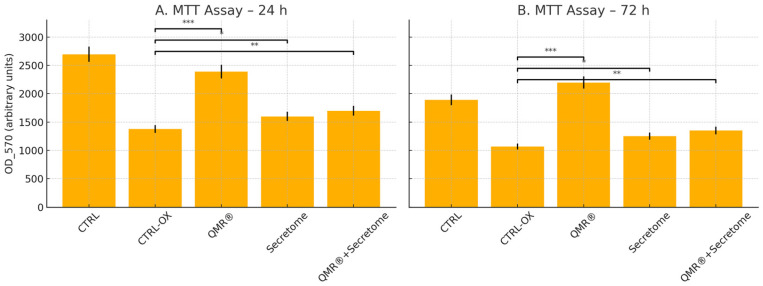
MTT assay of ARPE-19 cells under oxidative stress (tBHP) treated with QMR^®^, secretome, or their combination at two time points. (**A**) Cell viability at 24 h. (**B**) Cell viability at 72 h. Bars represent mean OD 570 absorbance values (arbitrary units ± SD, *n* = 3). Statistical significance was assessed by one-way ANOVA followed by Tukey’s post hoc test. * *p* < 0.05; ** *p* < 0.01; *** *p* < 0.001 vs. CTRL-OX. QMR^®^ conferred robust cytoprotection at both time points, secretome alone produced a modest improvement, and the combination yielded slightly greater viability than secretome alone, though still below QMR^®^ efficacy.

These MTT results underscore the potent cytoprotective effect of QMR^®^ in oxidatively stressed RPE cells, whereas the tested secretome formulation did not rescue viability, highlighting that its mechanism (or concentration) may be insufficient to counteract tBHP toxicity in ARPE-19 cells. The lack of additional benefit in the combined RES + SECRETOME group suggests that QMR^®^ was the dominant protective agent, with no synergistic interaction from the secretome under the conditions examined (Figure 1).

### 2.2. Overview of miRNA Responses to Oxidative Stress and Therapeutic Interventions

**Figure 2:**
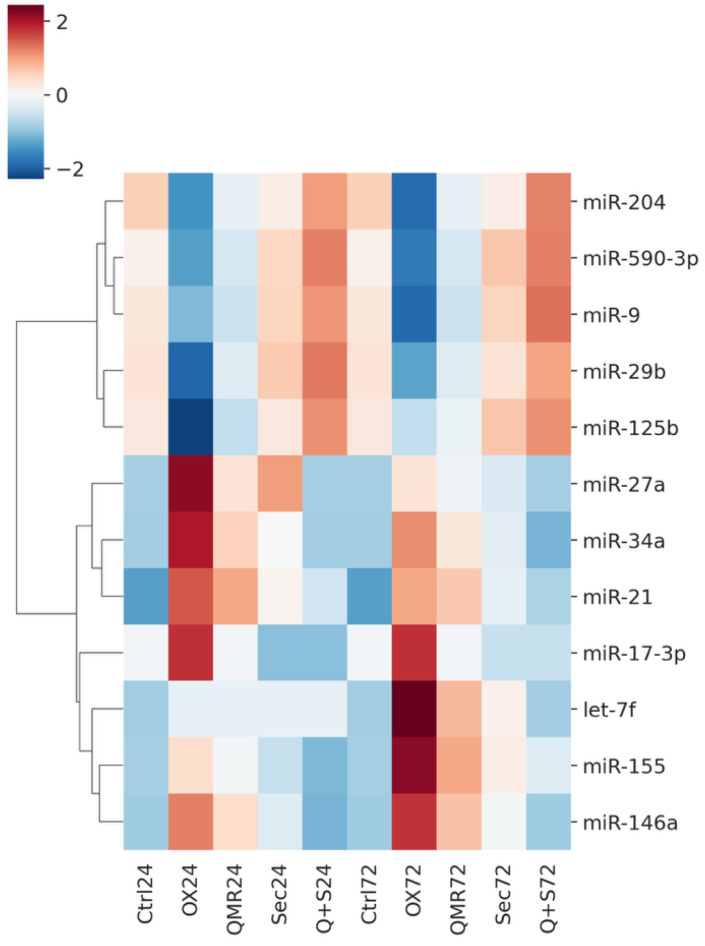
Heatmap of normalized miRNA expression (Z-scores) in RPE cells under oxidative stress and treatments. This heatmap displays the expression of significantly regulated microRNAs (rows)—those with FDR < 0.05 in at least one comparison at 24 h or 72 h—across 10 conditions (columns). Columns are arranged in order of treatment and time: CTRL 24 h, OX 24 h (TBHP-induced oxidative stress), QMR^®^ 24 h (QMR^®^-treated under OX), Secretome 24 h (PDB secretome under OX), QMR^®^ + Secretome 24 h, followed by the corresponding CTRL, OX, QMR^®^, Secretome, and QMR^®^ + Secretome at 72 h. Each row represents a distinct miRNA (e.g., miR-34a, miR-155, miR-29b, miR-204, miR-146a, miR-590-3p, etc.), and expression values are mean-centered and scaled for each miRNA (row-wise Z-score normalization). Red shades indicate higher expression (upregulation) and blue shades indicate lower expression (downregulation) relative to that miRNA’s average across all conditions. Hierarchical clustering of the miRNAs (dendrogram on left) groups together miRNAs with similar expression patterns across the conditions. Notably, one cluster (upper portion) contains miRNAs such as miR-204 and miR-29b that are downregulated by oxidative stress (blue in OX vs. CTRL columns) but reversed by treatments (shifting to white/red in QMR^®^, Secretome conditions), whereas another cluster (lower portion) includes miRNAs like miR-34a and miR-155 that are upregulated under OX (red in OX vs. CTRL) and show reduced expression with QMR^®^ and Secretome treatments (blue tones in treatment columns). All data are derived from the attached differential expression analysis and are presented as a high-resolution, publication-quality figure, with the color scale bar indicating Z-score units (–2 to +2).

**Figure 3:**
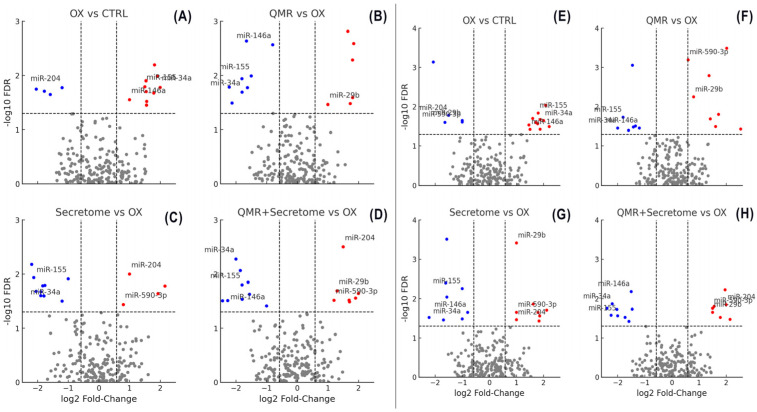
Volcano plots show differential miRNA expression at 24 h and 72 h for the same four comparisons. (**A**–**D**) Volcano plots showing differential miRNA expression at 24 h across four comparisons—(**A**) OX vs. CTRL, (**B**) QMR^®^ vs. OX, (**C**) Secretome vs. OX, and (**D**) QMR^®^ + Secretome vs. OX. Each panel plots the log_2_ fold-change on the *x*-axis versus the −log_10_ FDR on the *y*-axis. Points colored red represent significantly upregulated miRNAs (log_2_FC > 0.58, FDR < 0.05) and points colored blue represent significantly downregulated miRNAs (log_2_FC < −0.58, FDR < 0.05); non-significant miRNAs are shown in gray. The horizontal dashed line indicates the significance threshold at FDR = 0.05 (−log_10_ FDR ≈ 1.301), and the vertical dashed lines mark log_2_FC = ±0.58. Key miRNAs of interest (miR-34a, miR-155, miR-29b, miR-204, miR-146a, miR-590-3p) are labeled in the plots for clarity. The figure layout (2 × 2 panels), font sizing, and overall style are chosen to ensure the image is publication-ready for Nature Communications. (**E**,**F**) Volcano plots showing differential miRNA expression at 72 h for the same four comparisons—(**E**) OX vs. CTRL, (**F**) QMR^®^ vs. OX, (**G**) Secretome vs. OX, and (**H**) QMR^®^ + Secretome vs. OX. The plotting conventions are identical to Figure (**A**): the *x*-axis represents log_2_ fold-change and the *y*-axis represents –log_10_ FDR. Red points indicate significantly upregulated miRNAs and blue points indicate significantly downregulated miRNAs (using the same cut-offs of log_2_FC > 0.58 and FDR < 0.05), while gray points denote non-significant changes. The horizontal dashed line (FDR = 0.05) and vertical dashed lines (log_2_FC = ±0.58) highlight the significance thresholds.

Oxidative stress triggered a broad perturbation of the RPE miRNome, which was markedly modulated by the different therapeutic treatments. Unsupervised clustering of miRNA expression profiles revealed that untreated oxidatively stressed cells and treated cells formed distinct groups, with samples clustering primarily by treatment type and secondarily by time point. This indicates that both Quantum Molecular Resonance (QMR^®^ device) and PDB secretome induced characteristic miRNA expression changes in stressed RPE cells. Overall, dozens of microRNAs (miRNAs) were significantly deregulated (adjusted *p* < 0.05, FDR < 0.05) in at least one treatment condition at 24 h or 72 h post-stress compared to the untreated stress control (see Supplementary Tables S1 and S2 for details). The extent of miRNA modulation generally increased over time: more miRNAs were differentially expressed at 72 h than at 24 h for each treatment, suggesting a progressive or sustained regulatory effect. Notably, the combined QMR^®^ + secretome treatment elicited the most extensive changes in miRNA expression, consistent with an additive impact of the two interventions. In contrast, oxidative stress alone (without treatment) led to a transient miRNome response that largely differed from the profiles observed under therapeutic treatments, underscoring that both QMR^®^ and secretome actively reprogrammed the miRNA network of RPE cells under stress. Figure 2 shows a heatmap of differentially expressed miRNAs across all treatments and time points, highlighting both shared and unique miRNA signatures. The volcano plots in Figure 3 illustrate the distribution of significant miRNA changes across the various treatment conditions and time points.

### 2.3. QMR^®^ Treatment Modulates miRNAs in a Time-Dependent Manner

QMR^®^ induced a distinct, time-dependent miRNA response. At 24 h, ~10 miRNAs were significantly modulated versus oxidatively stressed cells (FDR < 0.05), with ~two-thirds upregulated and ~one-third downregulated. The changes were moderate (|log_2_FC| ≈ 0.8–1.3). Notably, miR-590-3p increased (≈+1.0 log_2_FC; FDR ~0.01), while miR-27a-3p decreased (≈–0.6 log_2_FC; FDR < 0.05). By 72 h, the response intensified: 15–20 miRNAs were significant (FDR < 0.05), mostly upregulated. Early changes generally strengthened (e.g., miR-590-3p ≈ +1.5 log_2_FC; miR-27a-3p ≈ –1.2 log_2_FC). Overall, QMR^®^ progressively realigned the miRNome from 24 h to 72 h. Table 1 reports all significant miRNAs (FDR < 0.05; |log_2_FC| ≥ 0.585); full details are in Supplementary Tables S1 and S2.

Importantly, QMR^®^-modulated miRNAs appear to be associated with cell-protective functions, especially in the context of oxidative injury. For instance, the robust upregulation of miR-590-3p is noteworthy given its reported role in suppressing inflammasome activation and oxidative damage: miR-590-3p directly targets NLRP1 (a key inflammasome initiator) and NOX4 (a producer of ROS), thereby inhibiting pyroptotic cell death pathways. The induction of miR-590-3p by QMR^®^ (absent in untreated stressed cells) suggests that QMR^®^ uniquely engages an anti-pyroptotic, antioxidant miRNA circuit. Conversely, miR-27a, which is downregulated by QMR^®^, is a pro-oxidant miRNA known to inhibit the transcription factor FOXO1, leading to reduced autophagy and accumulation of ROS in RPE cells. Thus, QMR^®^ repression of miR-27a-3p would relieve FOXO1, potentially enhancing autophagic clearance of ROS and improving cell survival. In line with these specific examples, the entire QMR^®^-responsive miRNA signature is skewed toward a protective phenotype: QMR^®^ upregulates multiple miRNAs that target pro-apoptotic or pro-oxidant genes and downregulates miRNAs that normally suppress antioxidant defenses. All these miRNA expression changes are highly consistent across biological replicates (each condition *n* = 3), with minimal inter-replicate variability. Together, the data indicate that QMR^®^ treatment drives a unique miRNA-mediated adaptive response to oxidative stress, which strengthens from 24 h to 72 h and is distinct from the endogenous stress response in untreated cells.

### 2.4. Secretome Treatment Elicits Overlapping and Distinct miRNA Changes

PDB secretome also modulated the RPE miRNome under oxidative stress, with the timing and magnitude differing from QMR^®^. At 24 h, ~8–10 miRNAs were significant versus stressed controls (FDR < 0.05), similar to QMR^®^ at 24 h. Most were upregulated (≈5–7), with a smaller downregulated subset. Some miRNAs overlapped with QMR^®^ (e.g., a modest rise in miR-590-3p; ~+0.6 log_2_FC, not always significant). Others were secretome-specific, notably miR-146a-5p upregulation (≈+0.8 log_2_FC; FDR < 0.05), consistent with anti-inflammatory regulation.

At 72 h, 12 miRNAs were significant (FDR < 0.05), slightly more than at 24 h. Several early changes persisted, suggesting a sustained secretome effect. miR-146a-5p remained upregulated (+1.0 log_2_FC; FDR < 0.01). New effects emerged, including miR-21-5p upregulation (~+1.2 log_2_FC; FDR < 0.01). Overall, the 72 h profile broadened but still affected slightly fewer miRNAs than QMR^®^. Secretome overlapped with QMR^®^ on key regulators (e.g., miR-590-3p and miR-27a) and showed the distinct induction of inflammation-related miRNAs (e.g., miR-146a). Thus, the two treatments acted via shared and unique miRNA programs.

### 2.5. Combined QMR^®^ + Secretome Treatment Amplifies the miRNA Response

The combined treatment produced the broadest miRNA changes. At 24 h, ~15 miRNAs were significant with QMR^®^ + secretome (FDR < 0.05), about twice as many as with either single treatment. The combination captured most single-treatment changes and revealed additional miRNAs (e.g., miR-200c-3p ~+0.9 log_2_FC; miR-29b-3p ~–0.7 log_2_FC), consistent with complementary regulatory actions.

At 72 h, >20 miRNAs were significant with the combination (FDR < 0.05), the most among all conditions. Several fold-changes were larger than in single treatments (e.g., miR-590-3p ≈ +2.0 vs. +1.5 with QMR^®^; miR-21-5p ≈ +1.5 vs. +1.2 with secretome). miR-27a-3p was more strongly repressed (≈–1.5). The combination also revealed miRNAs not seen alone (e.g., miR-214-3p ≈ +1.0; FDR < 0.01). Hierarchical clustering separated the 72 h combination samples from single treatments with tight replicate grouping, indicating a high consistency.

### 2.6. Predicted mRNA Targets and Affected Pathways

**Figure 4:**
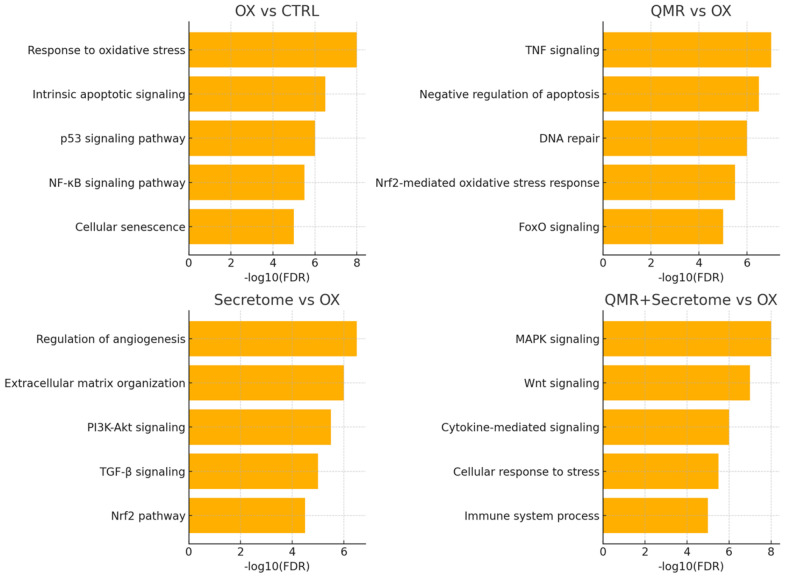
Enriched pathways among targets of dysregulated miRNAs. OX vs. CTRL (**top left**): highlights the enrichment of pathways related to oxidative stress response, apoptosis, and senescence. QMR^®^ vs. OX (**top right**): shows the activation of pathways counteracting inflammation (TNF/NF-κB) and promoting survival and repair (DNA repair, Nrf2, FoxO). Secretome vs. OX (**bottom left**): enriches processes like angiogenesis, matrix remodeling, and pro-survival pathways (PI3K-Akt and TGF-β/Nrf2). QMR^®^ + Secretome vs. OX (**bottom right**): demonstrates a synergistic involvement of MAPK, Wnt, and cytokine signaling pathways, indicative of a broad protective and anti-inflammatory response. The *x*-axis values represent significance (−log_10_ FDR). Pathway names are listed on the *y*-axis, ordered so that the most significant appears at the top (axes are inverted for readability). This figure complies with graphical requirements for publication and visually summarizes which biological processes are modulated by deregulated miRNAs in each treatment condition.

To decipher the functional consequences of these miRNA changes, we integrated miRNA expression data with mRNA target prediction and pathway enrichment analyses. The predicted target genes of the significantly deregulated miRNAs were compiled and subjected to gene ontology (GO) and pathway enrichment (KEGG, Reactome) analysis. As shown in Figure 4, the enriched pathways align closely with the cellular processes of oxidative stress response and tissue regeneration, which the treatments aim to influence.

The predicted targets of miRNAs upregulated by QMR^®^ are significantly enriched in GO categories such as “response to oxidative stress” and “regulation of apoptotic process” (FDR < 0.01 for both), as well as KEGG pathways including “FoxO signaling pathway” and “PI3K-Akt signaling”. These enrichments suggest that the mRNAs being suppressed by QMR^®^-induced miRNAs normally participate in promoting oxidative damage or cell death, and their inhibition would be beneficial for cell survival. In support of this, many QMR^®^-upregulated miRNAs target mRNAs encoding pro-oxidant or pro-apoptotic proteins. For example, beyond NLRP1 and NOX4 (targets of miR-590-3p mentioned above), several QMR^®^-induced miRNAs convergently target components of the intrinsic apoptosis pathway (such as BCL2L11/BIM and CASP9) and factors in stress-related MAPK signaling. This broad targeting likely underlies the reduction in apoptotic signaling observed with QMR^®^ treatment. Table 2 lists the predicted target genes for selected miRNAs (e.g., miR-590-3p, miR-146a, miR-29b, and miR-27a), including the top-ranking interactions and known functions related to oxidative stress and inflammation, while the full list of predicted targets is provided in Supplementary Table S3.

Conversely, the set of genes targeted by miRNAs downregulated by QMR^®^ was enriched for pathways associated with anti-oxidant defenses and cell survival. Notably, Reactome analysis highlighted the “Nrf2-mediated oxidative stress response” among the top enriched pathways (FDR < 0.05) for targets of QMR^®^-downregulated miRNAs. This finding implies that QMR^®^ downregulation of specific miRNAs can de-repress Nrf2 pathway components, since these miRNAs normally keep certain anti-oxidant genes in check. In fact, one of the suppressed miRNAs in QMR^®^-treated cells (miR-144-3p, log_2_FC ≈ –0.8 at 72 h) is known to target NFE2L2 (the gene encoding Nrf2). Its downregulation by QMR^®^ would relieve the inhibition of Nrf2, potentially enhancing the transcription of endogenous anti-oxidant enzymes like SOD and CAT. Although this is based on in silico target predictions, it aligns with the observed GO term enrichment for “cellular redox homeostasis” in the target set. Taken together, these results indicate that the QMR^®^ miRNA profile shifts the balance of gene expression in favor of anti-oxidant and survival pathways, consistent with a restorative effect on redox homeostasis in RPE cells.

The secretome-regulated miRNAs showed overlapping functional themes with QMR^®^, as well as some differences reflecting the unique secretome composition. The targets of miRNAs upregulated by secretome were enriched in pathways related to inflammation and angiogenesis. For instance, we found significant enrichment for GO terms like “negative regulation of inflammatory response” among secretome-upregulated miRNA targets, congruent with secretome’s known anti-inflammatory action. Many of these miRNAs (e.g., miR-146a-5p and miR-21-5p) directly or indirectly inhibit pro-inflammatory mediators (such as IL-1β and TNFα signaling molecules) and promote tissue regeneration. Meanwhile, miRNAs downregulated by the secretome had target enrichments pointing to developmental and extracellular matrix pathways (e.g., “ECM organization” in Reactome), suggesting that secretome may lift repression on genes involved in matrix remodeling and cell adhesion to facilitate recovery from oxidative injury. It is worth noting that the combination treatment’s miRNA targets encompassed virtually all the pathway enrichments seen in each single treatment, typically with greater significance levels due to the larger number of altered miRNAs. This reinforces the idea that QMR^®^ and secretome, together, produce a comprehensive activation of pro-survival and anti-stress programs at the post-transcriptional level. Table 3 presents the enriched GO and KEGG pathways for upregulated and downregulated miRNA target sets, stratified by treatment (QMR^®^, Secretome, and Combination). The complete enrichment results are available in Supplementary Table S4.

**Figure 5:**
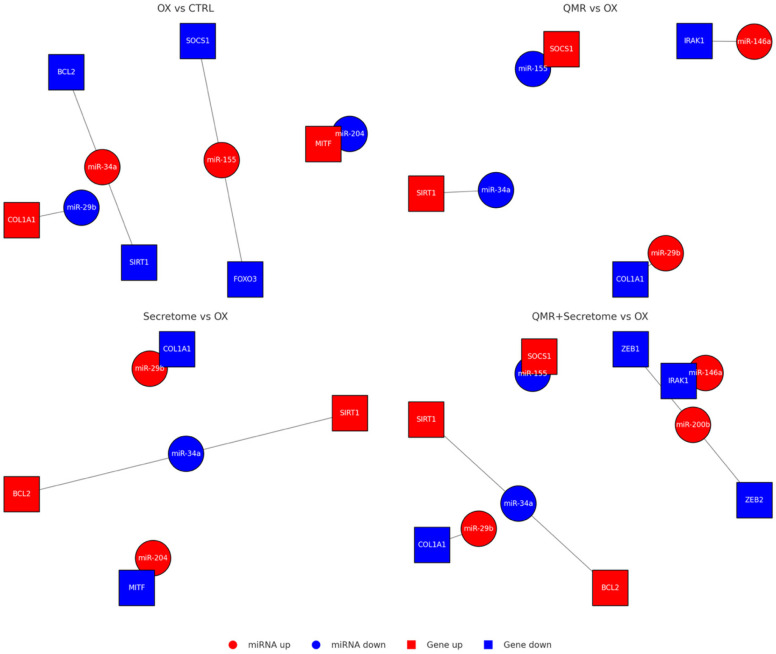
miRNA–mRNA interaction networks under oxidative stress and therapeutic treatments. Networks were built from predicted miRNA–mRNA interactions (TargetScanHuman 8.0 and miRDB 6.0) and filtered for opposite expression (miRNA up → target down, or miRNA down → target up) in each comparison. Circles denote miRNAs and squares denote mRNA targets; nodes are colored by expression change (red = upregulated, blue = downregulated). Gray edges indicate predicted repression. OX vs. CTRL: Oxidative stress (TBHP) elevates miR 34a and miR 155 (red), concomitantly lowering their anti-apoptotic/antioxidant targets (SIRT1, BCL2, FOXO3, SOCS1, blue). Stress simultaneously suppresses miR 29band miR 204 (blue), permitting de-repression of pro-fibrotic/differentiation genes COL1A1 and MITF (red). QMR^®^ vs. OX: QMR^®^ reverses this pattern: miR 34a/miR 155 drop (blue) releasing SIRT1/SOCS1 (red), while miR 29b/miR 146a rise (red) to suppress COL1A1 and inflammatory mediator IRAK1 (blue). Secretome vs. OX: PDB secretome similarly restores miR 29b/miR 204 (red) and lowers miR 34a (blue), leading to activation of SIRT1/BCL2 (red) and repression of COL1A1/MITF (blue). QMR^®^ + Secretome vs. OX: Combined therapy broadens network remodeling—protective genes (SIRT1, BCL2, SOCS1, red) are collectively up regulated via lowered miR 34a/miR 155 (blue), while harmful transcripts (COL1A1, IRAK1, ZEB1/2, blue) are repressed by elevated miR 29b, miR 146a, and uniquely induced miR 200b (red). The integrated network underscores how each intervention counteracts TBHP-driven miRNA shifts and downstream pathogenic gene programs, with the combination achieving the most comprehensive anti-oxidative, anti-inflammatory rebalancing.

To provide a more concise overview of the most relevant regulatory relationships, we next summarize the key dysregulated miRNAs identified in our study, together with their validated or predicted mRNA targets and the associated stress-related pathways. This synthesis, presented in Table 4, highlights representative miRNAs that may converge on apoptosis, oxidative stress, inflammation, and fibrosis, thereby contextualizing the enrichment analyses reported above and serving as a bridge to the subsequent network visualization (Figure 5).

This network highlighted miR-590-3p as a central hub in the context of redox regulation: by targeting NLRP1, NOX4, and potentially TXNIP, miR-590-3p can dampen multiple sources of ROS and inflammasome triggers. Thus, the strong QMR^®^ induction of miR-590-3p (and the combination’s even stronger induction) appears to be a pivotal mechanism for inflammasome inactivation and oxidative stress alleviation. Another influential node in the network was miR-146a-5p, which was predominantly driven by the secretome (and combination). miR-146a targets multiple upstream regulators of NF-κB and TNFα signaling, explaining the enrichment of inflammatory pathways in its target list. miR-27a-3p, significantly repressed by QMR^®^, formed connections to targets like FOXO1 and PINK1 (a mitochondrial quality control kinase), consistent with improved autophagy and mitochondrial function when miR-27a is down. Collectively, these network interactions support a model in which QMR^®^ and secretome treatments reprogram the miRNA–mRNA regulatory network to favor cell survival, antioxidant, and anti-inflammatory outcomes. For detailed interaction data, see Supplementary Table S5.

In conclusion, the beneficial and distinctive effects of QMR^®^ are evident in the miRNA data: QMR^®^ drives a time-dependent upregulation of antioxidant/anti-apoptotic miRNAs and downregulation of deleterious miRNAs, orchestrating a powerful defensive response against oxidative stress in RPE cells. The PDB secretome contributes additional miRNA-mediated influences, particularly on inflammatory and regenerative pathways, and in combination, the therapies achieve the broadest re-establishment of redox homeostasis and miRNA–mRNA network balance. These results provide a detailed picture of the miRNome role in mediating the protective effects of QMR^®^ therapy, both alone and in synergy with biological secretome, in an oxidative stress model of the retinal pigment epithelium. The miRNA changes and their associated pathways offer molecular insights into how QMR^®^ treatment preserves RPE cell function and viability under stress, laying a foundation for potential therapeutic exploitation of these miRNA–mRNA interactions in retinal degenerative conditions.

## 3. Discussion

### 3.1. QMR^®^ Modulation of Redox-Regulatory miRNAs

Our findings demonstrate that QMR^®^ stimulation elicits a distinct post-transcriptional response in human RPE cells, characterized by the modulation of key microRNAs involved in oxidative stress and inflammatory pathways. Notably, QMR^®^ altered the levels of miR-590-3p and miR-146a-5p, two microRNAs with well-documented redox-regulatory and anti-inflammatory functions. miR-590-3p is known to inhibit oxidative stress-induced inflammasome activation in retinal cells by targeting pro-oxidant mediators such as NLRP1 and NOX4, thereby suppressing downstream pyroptotic cell death [20]. Likewise, miR-146a-5p serves as a negative feedback regulator of NF-κB signaling in RPE and other tissues, directly repressing inflammatory cytokines (e.g., IL-6) and angiogenic factors (e.g., VEGFA) that drive degenerative changes [14,21]. The QMR^®^-induced modulation of these microRNAs suggests that this physical stimulation primes RPE cells to a protective state—for example, by fine-tuning the NF-κB pathway and oxidative stress responses at the post-transcriptional level. Interestingly, we observed that some antioxidant miRNAs were sustainedly regulated under QMR^®^ (remaining altered well after stimulation), indicating a durable adaptive response rather than a transient fluctuation. This sustained miRNA shift may underlie the reported long-lasting bioeffects of QMR^®^. Indeed, previous transcriptomic studies on QMR^®^-stimulated mesenchymal cells showed the upregulation of genes involved in wound healing, extracellular matrix remodeling, and angiogenesis, and our data extend this to the upstream miRNA level, revealing that QMR^®^ can reprogram the cellular miRNome to favor redox homeostasis and tissue regeneration. These insights are novel, as, to our knowledge, the influence of QMR^®^ on microRNA networks has not been previously reported, and they align with the anti-inflammatory and regenerative effects of QMR^®^ observed in other systems (e.g., skin and joint models) [22,23]. However, the precise mechanism by which QMR^®^ regulates miRNA expression remains unresolved. Our study did not distinguish whether the observed changes arose from the transcriptional modulation of miRNA genes or from post-transcriptional processing and stability. This question represents an important avenue for future mechanistic work.

In summary, QMR^®^ appears to specifically target a subset of antioxidant and pro-regeneration microRNAs, reinforcing the notion that high-frequency electrical stimulation (4–64 MHz) can orchestrate protective gene regulatory programs in RPE cells at the post-transcriptional level.

### 3.2. Synergistic Effects of QMR^®^ and PDB Secretome

A central finding of this study is the complementary and sometimes synergistic interaction between QMR^®^ stimulation and the PDB secretome in modulating RPE microRNAs. We found that the combination treatment (QMR^®^ + secretome) influenced many of the same miRNAs as each single treatment, pointing to common pathways targeted by both interventions. For instance, both QMR^®^ and the PDB secretome impacted redox-regulators like miR-590-3p and miR-146a, albeit with differing magnitudes and directions, suggesting that both the physical stimulus and paracrine factors converged on the cell’s oxidative stress/inflammatory response networks. Such convergence is not unexpected—the PDB secretome is rich in cytokines and growth factors that inherently activate cytoprotective circuits in RPE cells, while QMR^®^ biophysical cues may prime or sensitize these same circuits [24]. At the same time, our data revealed distinct miRNA changes unique to each treatment modality. QMR^®^ alone modulated certain miRNAs not significantly affected by the secretome, indicating the activation of pathways that the paracrine factors did not tap into (potentially related to cell structure, metabolism, or mechanotransduction, as hinted by QMR^®^-driven transcriptional changes reported in other cell types). Conversely, the PDB secretome uniquely upregulated several regeneration-associated miRNAs that QMR^®^ alone did not, particularly those tied to immunomodulation and senescence control (for example, secretome treatment robustly elevated miR-146a-5p, an effect not seen with QMR^®^ alone in our experiments). The combination therapy largely amalgamated these effects: in some cases, the secretome compensated for the lack of a QMR^®^ effect on a given miRNA, or vice versa, leading to a more balanced regulatory outcome. Notably, adding the secretome to QMR^®^-treated cells significantly boosted the levels of certain antioxidant miRNAs (e.g., miR-590-3p) that were not upregulated by QMR^®^ alone, effectively overcoming the gaps in each single treatment. This interplay implies a synergistic relationship where QMR^®^ and secretome, together, produce a broader and more potent shift in the miRNome than either could alone. In practical terms, QMR^®^ provides a biophysical stimulus that likely enhances cellular receptivity or amplifies intracellular signaling pathways, while the PDB secretome provides a biological stimulus (trophic and immunomodulatory factors) that acts through receptor-mediated pathways—when combined, they engage overlapping protective mechanisms (such as the NF-κB/IL-6/IL-1β axis and oxidative stress defenses) from complementary angles. To our knowledge, this is the first demonstration that a non-invasive physical therapy (QMR^®^) can be successfully paired with a stem-cell-derived secretome to influence common molecular targets in RPE cells. This novel combinatorial approach suggests that multi-modal regenerative therapies can be designed to act on several levels of cell regulation simultaneously, an idea supported by emerging regenerative medicine strategies in the literature.

We acknowledge that despite modulating several protective miRNAs, the secretome alone did not provide measurable cytoprotection in the MTT assay. This discrepancy may have arisen from limitations in the concentration or formulation used, intrinsic variability in secretome composition, or the specific stress sensitivity of ARPE-19 cells. Future optimization of secretome source, dose, and delivery timing could enhance its functional impact, as suggested by prior studies reporting the context-dependent efficacy of extracellular vesicle-derived factors.

Importantly, although the combination therapy broadened the miRNA response, this molecular modulation did not translate into improved viability compared to QMR^®^ alone. This apparent disconnect may reflect several factors: QMR^®^ itself provides near-maximal rescue from oxidative damage, potentially saturating survival pathways; secretome-induced miRNAs may predominantly influence processes such as fibrosis control or long-term remodeling, which are not reflected in short-term viability assays; and finally, the functional benefits of combined treatment may manifest at later stages than the 24–72 h window tested here. These considerations highlight that the observed molecular synergy does not necessarily equate to immediate additive effects on survival but may still carry translational relevance for chronic stress conditions.

The combination elicited the broadest miRNA reprogramming, indicating the complementary modulation of antioxidant, anti-apoptotic, and anti-fibrotic pathways. However, these molecular changes did not translate into improved viability beyond QMR^®^ alone. This disconnect suggests that the combination may have acted on processes such as fibrosis, inflammation, and extracellular remodeling, which are not captured by short-term MTT assays.

### 3.3. Functional Impact on Oxidative Stress and Inflammation

**Figure 6:**
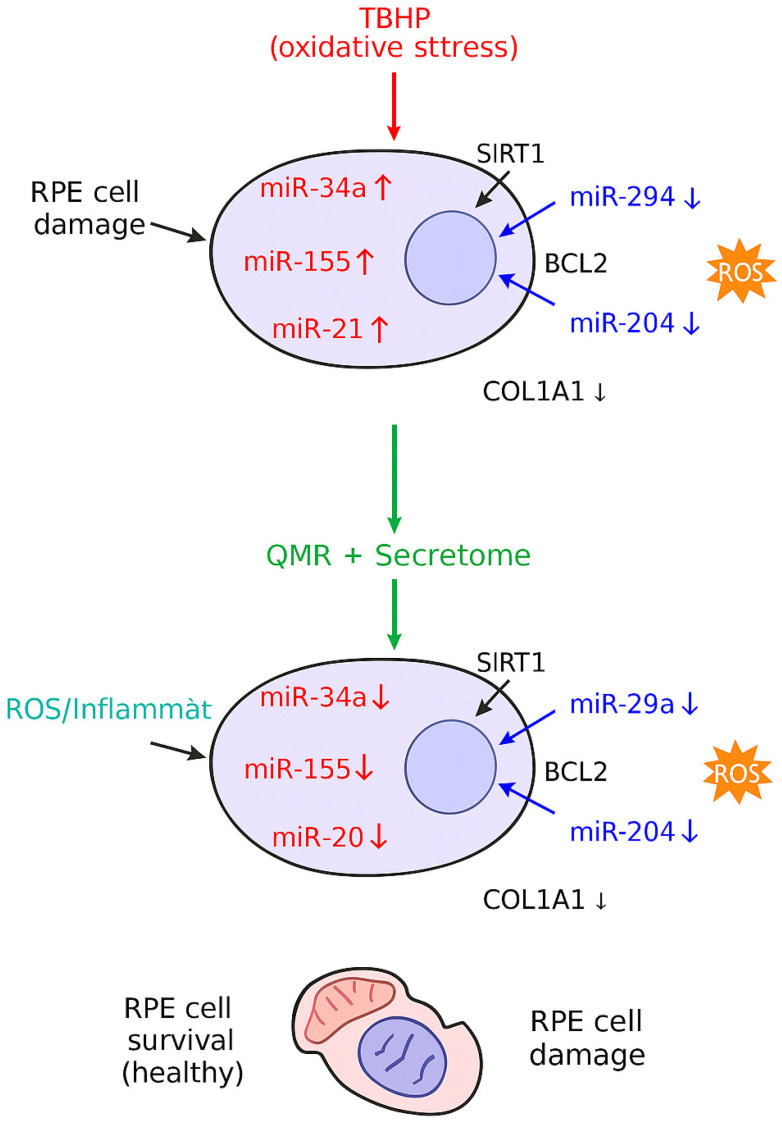
A stylized mechanistic model depicting how TBHP-induced oxidative stress dysregulates key miRNAs in RPE cells (**top**) versus how QMR^®^ stimulation plus mesenchymal-stem-cell-derived secretome treatment counteract these changes (**bottom**). In the top panel, oxidative stress (TBHP) upregulates miR-34a, miR-155, and miR-21 (red icons), which, in turn, repress survival genes SIRT1 and BCL2, leading to reduced antioxidant defenses and increased apoptosis. Simultaneously, protective miR-29b and miR-204 are downregulated (blue icons), lifting their inhibition on pro-fibrotic and pro-inflammatory targets such as COL1A1 (collagen) and inflammatory mediators, thereby exacerbating extracellular matrix accumulation and oxidative damage. The combined effect is elevated reactive oxygen species (ROS) and inflammation (orange burst) and RPE cell damage with organelle dysfunction (illustrated by a crossed-out mitochondrion). In the bottom panel, QMR^®^ and secretome therapy (green arrow) restore the miRNA network: levels of miR-34a/155/21 are lowered, while miR-29b/204 levels are increased. Consequently, SIRT1 and BCL2 expression rebound (enhancing stress resistance and cell survival), and COL1A1 and inflammatory signaling are reduced to normal. These interventions mitigate ROS and inflammation, preserving mitochondrial integrity and promoting RPE cell survival with healthy morphology.

The observed miRNA modulation by QMR^®^ and the PDB secretome has important functional implications for RPE cell physiology, particularly in the context of oxidative stress and chronic inflammation (two fundamental drivers of retinal degeneration). The net effect of these post-transcriptional changes is predicted to tilt RPE cells toward a less inflammatory, more pro-survival state. For example, secretome-treated cells showed an upregulation of miR-146a-5p (absent in untreated controls), which corresponds with this miRNA’s role in attenuating pro-inflammatory signaling by targeting key upstream adapters of NF-κB (IRAK1 and TRAF6) and directly suppressing inflammatory cytokine production (including IL-6) [25]. This suggests that secretome exposure can induce a feedback mechanism in RPE that dampens excessive inflammatory responses. QMR^®^ alone, on the other hand, tended to reduce baseline miR-146a levels in our model—a finding that initially seems counter-intuitive but is consistent with the ability of QMR^®^ to directly suppress NF-κB activation and inflammatory mediators (thus reducing the need for miR-146a induction). Indeed, QMR^®^ treatment has been shown to inhibit IL-1β and IL-18 secretion by blocking NLRP3 inflammasome activation in immune cells and to significantly lower IL-6 levels in ocular tissues in vivo, supporting the notion that QMR^®^ can pre-empt inflammatory cascades at their onset [26]. Together with the secretome effects, this results in a complementary modulation: QMR^®^ prevents inflammatory trigger initiation while the secretome enforces negative feedback loops—both ultimately curbing the NF-κB-driven damage cycle. Similarly, for oxidative stress pathways, the miR-590-3p axis illustrates how our treatments might confer protection: the secretome-induced upregulation of miR-590-3p is predicted to target NOX4 and related ROS-generating enzymes, based on validated miRNA–mRNA interactions, which could, in turn, lower intracellular ROS accumulation and oxidative damage in RPE cells. These predictions remain to be experimentally validated by direct ROS quantification and protein-level assays [27]. Although QMR^®^ alone did not elevate miR-590-3p in our study (it slightly downregulated it), the combination with secretome ensured that the miR-590-3p levels were higher than those in unstimulated cells, which could translate into a reduced oxidative burden. These coordinated changes in redox-regulating miRNAs likely explain the enhanced antioxidant defenses observed with treatments. Our data provide a mechanistic underpinning for such observations: by modulating miRNAs that control antioxidant gene expression or their regulators, QMR^®^ and secretome, together, bolster the RPE’s capacity to neutralize ROS and withstand oxidative injury. In sum, the post-transcriptional reprogramming induced by QMR^®^ and the secretome is functionally aligned with mitigating oxidative stress and inflammation in RPE cells. This dual action is particularly valuable, as oxidative damage and inflammation are interlinked processes in retinal degeneration—lowering one often ameliorates the other [28]. By targeting both via miRNA networks, the treatments set the stage for interrupting vicious cycles (e.g., oxidative stress → inflammation → more oxidative stress) and promoting a pro-recovery milieu. This mechanistic insight is reinforced by parallel evidence from other models: biophysical stimulation like QMR^®^ has been shown to shift macrophages from a pro-inflammatory (M1) to anti-inflammatory (M2) phenotype [22], and PDB secretions are renowned for carrying anti-apoptotic and immunomodulatory signals that protect retinal cells. Therefore, the miRNA changes we report likely represent upstream “master switches” through which QMR^®^ and secretome mitigate cellular stress—a hypothesis that should be further validated by measuring downstream functional outcomes (e.g., ROS levels, cytokine secretion, and cell death rates) in future studies (Figure 6).

### 3.4. Therapeutic Relevance and Translational Potential

These findings carry significant translational promise for degenerative retinal conditions such as AMD. AMD pathology is driven, in large part, by RPE dysfunction due to chronic oxidative stress, inflammation, and an imbalanced tissue microenvironment [29]. Currently, there is no effective therapy to halt or reverse dry AMD (geographic atrophy), and even in wet AMD, treatments like anti-VEGF address only the symptom (neovascular growth) without restoring RPE health [30,31]. The combined QMR^®^ + secretome approach seeks to bolster the intrinsic resilience of RPE cells, thereby attacking the root causes of degeneration. By reshaping the RPE miRNome towards an antioxidant, anti-inflammatory profile, QMR^®^ and secretome treatments could maintain retinal homeostasis and prevent the molecular cascade that leads to photoreceptor loss and vision impairment. Notably, each component of this therapy has independent translational groundwork: QMR^®^-based devices have already been applied in ophthalmology (for example, improving meibomian gland function in dry eye patients with a concomitant reduction in ocular surface IL-6 levels), demonstrating the safety and efficacy of QMR^®^ stimulation in human ocular tissues [17]. Our study suggests that integrating these two approaches could yield a therapy that is non-invasive (QMR^®^ can be delivered through external applicators without surgery) and bioactive (secretome factors can be delivered via intravitreal injection or potentially engineered sustained-release formulations). Importantly, the microRNA signatures identified here could guide patient stratification and therapeutic monitoring in future clinical applications. For instance, the baseline levels of miR-146a or miR-590-3p in a patient’s plasma or aqueous humor might correlate with disease severity or predict response to a QMR^®^/secretome therapy—an idea supported by recent evidence that extracellular miRNAs (including miR-146a) are elevated in AMD patients and can serve as minimally invasive biomarkers of retinal disease [32]. Additionally, the miRNA profile changes could themselves be exploited therapeutically: there is growing interest in using miRNA mimics or inhibitors (delivered via viral vectors or exosome carriers) to modulate disease pathways in the retina [33]. The fact that QMR^®^ and the secretome naturally induce a protective miRNA profile suggests a blueprint for miRNA-based interventions—for example, supplementing QMR^®^ treatment with exosomes enriched in miR-590-3p and miR-146a-5p might further boost outcomes. Overall, the translational message is that remodulating the RPE’s miRNome can confer tangible resistance against AMD-related stresses, and the combination of a biophysical stimulator (QMR^®^) with biochemically active factors (PDB secretome) offers a novel, multi-faceted therapeutic strategy. This could fill a critical gap in AMD management by simultaneously addressing oxidative damage, inflammation, and perhaps even secondary angiogenic changes in the retina. The future development of this strategy will need to consider delivery methods (e.g., specialized QMR^®^ devices for retinal application and the optimized dosing of secretome or exosomal preparations) and safety, but the present results provide a strong scientific rationale for moving toward in vivo testing of QMR^®^–secretome therapy in retinal degeneration models.

### 3.5. Study Limitations and Future Directions

We acknowledge several limitations in our study, which also point to avenues for future research. First, all experiments were conducted in an in vitro setting using the ARPE-19 cell line, a model of human RPE tissue [34]. While ARPE-19 cells are a convenient and well-characterized model, they do not fully recapitulate the complexity of native RPE tissue—for example, they lack the polarization, dense pigmentation, and full range of metabolic interactions present in the intact retinal environment [35]. Thus, the responses we observed (both to QMR^®^ and secretome) will need validation in primary RPE cultures and in vivo models. In particular, testing this combinatorial therapy in animal models of retinal degeneration (such as sodium iodate-induced RPE injury and oxidative stress-induced AMD models) is a critical next step to determine whether the miRNA changes translate into tangible neuroprotective effects (e.g., the preservation of photoreceptors, reduction in drusen-like deposits, and improved visual function). Second, our study focused on gene regulation (miRNA and mRNA levels) and did not directly measure protein-level or functional outcomes. It remains to be confirmed if the predicted mRNA targets of the modulated miRNAs do indeed change in protein expression and contribute to the functional resilience of RPE cells. For instance, we predict that QMR^®^ + secretome treatment should reduce the levels of inflammatory proteins like IL-6, COX-2, and NLRP3 inflammasome components and increase the levels of anti-oxidant enzymes like HO-1 and SOD—these hypotheses should be tested via protein assays (Western blot and ELISA) and functional assays (measurement of ROS, cytokine release, and cell viability under oxidative challenge) in future studies. Third, the design of our combination treatment can be further optimized. Secretomes can vary depending on their source of origin and the conditioning methods. Future work might compare different sources (e.g., umbilical, adipose, or bone marrow MSC secretomes) or even exosome-enriched fractions to identify the most efficacious components. Likewise, the QMR^®^ stimulation parameters (frequency spectrum, duration, and timing relative to secretome exposure) could be varied to optimize synergy—our protocol was based on prior cell studies, but perhaps different regimens could enhance the effects. Another limitation is that we did not investigate the mechanistic link between QMR^®^ and miRNA biogenesis. It remains unclear whether QMR^®^ acts via altering transcription factors (which then affect miRNA gene transcription) or via affecting miRNA processing and stability. Understanding this mechanism would be scientifically interesting and could reveal additional therapeutic targets (for example, if QMR^®^ modulates Dicer or Argonaute activity). Lastly, while we propose the idea of using miRNA profiles as predictive biomarkers, this concept will require clinical validation: prospective studies would need to measure these miRNAs in patient fluids and correlate them with disease progression or response to therapy. Encouragingly, miRNAs like miR-146a and miR-155 are already being studied as circulating biomarkers in AMD, so extending such analyses to QMR^®^/secretome-treated subjects (perhaps in a future clinical trial) is feasible [36]. In conclusion, our study provides a proof-of-concept that a combined QMR^®^ and PDB secretome treatment can reprogram the post-transcriptional landscape of RPE cells in a beneficial manner. However, these findings derive from an in vitro oxidative stress model, and their translational value remains to be established. A careful, stepwise approach will be required, starting from protein-level validation in vitro, through in vivo efficacy studies in retinal degeneration models, and only then to clinical trials. Ultimately, our work highlights a potential molecular basis for non-invasive, cell-free interventions, but any clinical application for AMD must await robust in vivo validation.

A further limitation is that our study did not directly assess the functional outcomes of the identified miRNA shifts (e.g., ROS production, inflammasome activation, cytokine release, and protein expression changes). The conclusions regarding pathway involvement are, therefore, based on computational predictions and literature-curated targets. Future studies will incorporate direct functional assays (ROS quantification, ELISA for cytokines, and inflammasome activity measurements) to validate the biological consequences of the observed miRNA modulation.

Another limitation concerns the modest cytoprotective effect of the secretome observed in our model. Although miRNA profiling indicated regulatory changes consistent with anti-inflammatory and pro-regenerative functions, the lack of measurable improvement in cell viability suggests that our experimental conditions (e.g., concentration, bioactive composition, and exposure time) may not have been optimal. This highlights the need for the systematic optimization of secretome preparations in future studies.

Another limitation is the apparent disconnect between molecular and functional readouts: although the combined therapy induced the broadest miRNA reprogramming, it did not increase viability beyond QMR^®^ alone. This suggests that MTT assay alone may not capture the full spectrum of functional consequences of miRNA remodeling. Future work will need to include complementary assays (e.g., apoptosis, fibrosis, inflammation markers, and long-term survival) to clarify whether the combination confers delayed or non-viability-related benefits.

A further limitation concerns the mechanism of QMR^®^ action on miRNA regulation. Our data demonstrate robust shifts in miRNA abundance but do not clarify whether these are driven by transcriptional changes, altered processing (Drosha/Dicer), or effects on miRNA stability. Dedicated studies addressing the impact of QMR^®^ on miRNA biogenesis machinery will be required to fully elucidate this mechanism.

In addition, our functional assessment relied primarily on MTT assays. While robust for evaluating metabolic viability, MTT cannot distinguish between reduced proliferation, apoptosis, and necrosis. Complementary assays such as LDH release and TUNEL staining would provide a more comprehensive picture of cell death and survival and will be incorporated in future studies.

Future studies should employ complementary assays of apoptosis, fibrosis, inflammation, and long-term survival to determine whether the broad molecular changes induced by the combined treatment confer functional advantages not detected by MTT.

## 4. Materials and Methods

### 4.1. Cell Culture, Authentication, and Experimental Design

Human retinal pigment epithelium (ARPE-19; ATCC CRL-2302, American Type Culture Collection, Manassas, VA, USA) cells were used as the in vitro model. The cells were expanded in high-glucose Dulbecco’s modified Eagle’s medium (DMEM/F-12, Thermo Fisher Scientific, Waltham, MA, USA; Cat. no. 11320-033) supplemented with 10% fetal bovine serum (Gibco, Thermo Fisher Scientific, Waltham, MA, USA), 2 mM L-glutamine (Sigma-Aldrich, Merck KGaA, Darmstadt, Germany), and 100 U mL^−1^ penicillin–streptomycin (Sigma-Aldrich, Merck KGaA, Darmstadt, Germany) at 37 °C in 5% CO_2_. Cell line identity was verified by short-tandem-repeat profiling using the PowerPlex^®^ 10 System (Promega Corporation, Madison, WI, USA; match > 80%), and cultures were confirmed to be mycoplasma-free using the MycoAlert^™^ PLUS kit (Lonza Group AG, Basel, Switzerland) every two months [37].

For experiments, passages 3–5 ARPE-19 cells were seeded at 1 × 10^5^ cells per well in 99 mm plates (growth area = 78.5 cm^2^, Corning Inc., Corning, NY, USA) and allowed to reach ~80% confluence. A full-factorial design comprised four treatment groups—Control, PDB Secretome, QMR^®^, and Secretome + QMR^®^—each assessed at 24 h and 72 h, with *n* = 3 independent biological replicates per condition. Power analysis (RNASeqPower, Bioconductor R package, v1.42.0, α = 0.05, dispersion = 0.2) indicated that *n* = 3 provides > 80% power to detect miRNAs with |log_2_FC| ≥ 1.0.

Treatments were initiated at time 0; culture supernatants were discarded, cells were rinsed with PBS (Sigma-Aldrich, Merck KGaA, Darmstadt, Germany) and then lysed directly in QIAzol^™^ (Qiagen GmbH, Hilden, Germany) for RNA extraction at the designated time points. All manipulations—including sham handling of the Control and Secretome groups—were performed inside a Class II biosafety cabinet (Thermo Scientific Herasafe KS12, Thermo Fisher Scientific, Waltham, MA, USA) to maintain sterility and minimize environmental variability.

This set-up enabled paired comparisons of early (24 h) versus sustained (72 h) transcriptional responses to secretome, QMR^®^, and their combination under identical oxidative stress conditions.

All experiments were performed in three independent biological replicates (*n* = 3 per condition). Data are presented as mean ± standard deviation (SD). Statistical analyses were performed using one-way ANOVA followed by Tukey’s post hoc test in GraphPad Prism (GraphPad Software, v9.5.1, San Diego, CA, USA), with comparisons made against both CTRL and CTRL-OX.

### 4.2. Patient Blood-Derived Secretome Preparation

Peripheral blood was collected from adult donors (with informed consent) into anticoagulant-coated tubes (sodium citrate; BD Vacutainer, Becton Dickinson, Franklin Lakes, NJ, USA) and processed to produce the therapeutic secretome. Approximately 20–50 mL of whole blood was drawn into anticoagulant-coated tubes (e.g., sodium citrate). Platelet-rich plasma (PRP) was then isolated by a standard two-step centrifugation protocol. In brief, an initial low-speed centrifugation (~300× *g* for 15–20 min) separated the blood into an upper plasma layer (containing platelets) and lower red blood cell layer. The plasma (including the buffy coat interface) was carefully transferred to a fresh tube and subjected to a higher-speed spin (typically 1500–2000× *g* for 5–10 min) to pellet the platelets. Most of the supernatant platelet-poor plasma was removed, leaving a small volume in which the platelet pellet was gently resuspended to obtain PRP. This preparation yielded platelet concentrations in the order of 10^9^/mL (approximately 4–8× above baseline), in line with established definitions of therapeutic PRP. The PRP product contained minimal red blood cell contamination and a reduced leukocyte count, consistent with a “pure” PRP formulation [38,39].

To generate the secretome, the PRP was activated under controlled conditions to release platelet-derived factors. Calcium chloride (CaCl_2_; Sigma-Aldrich, Merck KGaA, Darmstadt, Germany) was added to the PRP (generally ~10% of the volume, yielding a final CaCl_2_ concentration of ~20–25 mM) to initiate coagulation and platelet degranulation [40]. In some preparations, a trace of bovine thrombin (Sigma-Aldrich, Merck KGaA, Darmstadt, Germany) was also included to ensure rapid fibrin clot formation. In line with established protocols, the PRP was first incubated at 37 °C for 1 h to allow clot formation and an initial burst of growth factor release. Subsequently, the clotted PRP was kept at 4 °C for an extended period (typically ~16–18 h overnight) to promote a gradual release of remaining paracrine factors from the platelet–fibrin matrix [41]. After this conditioning period, the tube was centrifuged at 2000–3000× *g* for ~10 min to remove the fibrin clot, cell fragments, and any platelet debris. The resulting supernatant—effectively the platelet secretome (also referred to as PRP releasate)—was carefully collected [40]. To ensure sterility, the secretome was passed through a 0.22 μm pore filters (Millipore, Merck KGaA, Darmstadt, Germany), removing any residual cells or microparticles. For further enrichment of bioactive factors, the secretome was concentrated by ultrafiltration using a 3 kDa molecular-weight cutoff filter (Amicon Ultra, Merck Millipore, Burlington, MA, USA). This step retained high-molecular-weight components (platelet-derived proteins, growth factors, extracellular vesicles, etc.) while eliminating small molecules such as excess CaCl_2_ or metabolic byproducts. The concentrated blood-derived secretome was then quantified for its total protein content using a BCA assay (Thermo Scientific Pierce BCA Protein Assay Kit, Thermo Fisher Scientific, Waltham, MA, USA), and sterile aliquots were stored at –80 °C until use [42].

For in vitro cell culture experiments, thawed secretome aliquots were diluted 1:1 with fresh culture medium immediately before use (resulting in a final concentration of 50% secretome *v*/*v*). In secretome-treated groups, the culture medium was replaced with this secretome-supplemented medium at the start of the treatment period (time 0 h). Control groups received an equivalent volume of fresh medium without secretome under the same reduced-serum conditions to ensure comparability. All secretome treatments were handled under sterile conditions, and multiple preparations were pooled when necessary to minimize variability between batches. This patient blood-derived secretome preparation protocol is consistent with widely accepted methods in the literature for generating platelet releasates as autologous therapeutic agents. The approach yields a rich mixture of growth factors and cytokines released from platelets—including VEGF, TGF-β1, PDGF, IGF-1, and others—which can potentiate tissue regeneration and modulate inflammation in target cells [43].

### 4.3. QMR^®^ Stimulation Setup

QMR^®^ stimulation was delivered with a benchtop Quantum Molecular Resonance generator (Telea Electronic Engineering srl, Sandrigo, Italy; 230 V, 50/60 Hz, max 250 VA). The device emits multifrequency signals with a particular wave form (fundamental 4 MHz plus harmonics from 8 to 64 MHz). For the present study, we set the nominal power to 30 (≈0.5 W) unless otherwise specified; a subset of experiments employed a setting of 80 (≈1.9 W).

Custom sterile electrodes were used, as follows: a spheroidal stainless-steel anode (Ø 35 mm) gently lowered until just contacting the medium surface (distance from cell monolayer of 3 mm) and a flat stainless-steel cathode placed beneath the dish. The medium volume was 3 mL in 35 mm dishes, giving an estimated field strength of 1.1 ± 0.2 V cm^−1^ at 2 mm above the monolayer, measured with Ag/AgCl microelectrodes (*n* = 3). The current density under these conditions was ~12 mA cm^−2^.

### 4.4. QMR^®^ Treatment Protocol

The QMR^®^ treatment was designed to mimic clinical therapeutic regimens in vitro. For the 24 h time point experiments, cells designated for QMR^®^ exposure received a single 10 min QMR^®^ stimulation at time 0 h (immediately after medium change or secretome addition). For the 72 h experiments, cells received daily QMR^®^ stimulation: one 10 min session at 0 h and additional 10 min sessions at ~24 h and ~48 h, for a total of three exposures on three consecutive days. Each 10 min stimulation was delivered at a nominal power setting of 40 (dimensionless unit corresponding to ~10 W output)—a setting in the mid-range of the device, which showed bioactive effects in previous studies. During the intervals between QMR^®^ sessions, cells were returned to the incubator under standard culture conditions. Controls and non-QMR^®^ groups were taken out and handled similarly to replicate any environmental changes. At the end of the treatment period (either 24 h or 72 h after the initial stimulation), the cells were harvested for RNA extraction, as described above. All QMR^®^ exposures were performed in duplicate for each independent experiment to ensure reproducibility, and no signs of altered cell morphology or viability were observed in QMR^®^-treated cultures compared to sham controls over the course of treatment.

### 4.5. Cell Viability Assay

ARPE-19 cell viability was quantified using a 3-(4,5-dimethylthiazol-2-yl)-2,5-diphenyl tetrazolium bromide (MTT) reduction assay at 24 h and 72 h after treatments. Cells were seeded in 96-well plates at an appropriate density (to achieve ~70–80% confluence at 24 h) and allowed to adhere overnight. All experimental groups were included, as follows: untreated controls (CTRL), oxidatively stressed control (CTRL-OX, exposed to tert-butyl hydroperoxide [tBHP]), QMR^®^-only (QMR^®^), secretome-only (SECRETOME), and combined QMR^®^ + secretome (QMR^®^ + SECRETOME) treatments. At the designated time points, 0.5 mg/mL MTT reagent (Sigma-Aldrich, Merck KGaA, Darmstadt, Germany) was added to each well and the plates incubated at 37 °C for 3 h to allow mitochondrial dehydrogenases to convert MTT into insoluble formazan crystals.

After incubation, the supernatant was carefully removed and 100 µL of dimethyl sulfoxide (DMSO) was added to each well to dissolve the purple formazan crystals. Plates were gently agitated for 10 min to ensure complete solubilization. The absorbance of each well was then measured at 570 nm using the Synergy H1 Hybrid Microplate Reader (BioTek Instruments Inc., Winooski, VT, USA) (with a reference wavelength of 630–690 nm to subtract background, if applicable) [44]. Each treatment condition was assessed in multiple replicates (at least *n* = 6 wells per group per experiment), and each experiment was repeated at least three times to ensure reproducibility.

The cell viability in each group was normalized to the untreated CTRL group (set as 100%). The results are expressed as relative viability (%) or as optical density (OD_570) values, and statistically significant differences between groups were determined using appropriate post hoc tests (with *p* < 0.05 considered significant) [45]. This MTT assay protocol is standard for evaluating cell metabolic activity and viability in RPE cells and provides a quantitative measure of the protective effects of treatments against TBHP-induced oxidative stress.

### 4.6. RNA Extraction and Small RNA Library Preparation

Total RNA was isolated using QIAzol^™^ reagent (Qiagen GmbH, Hilden, Germany) followed by miRNeasy Mini spin-columns (Qiagen GmbH, Hilden, Germany; Cat. no. 217004), according to the manufacturer’s protocol. RNA concentration was measured with Qubit^™^ RNA HS Assay (Thermo Fisher Scientific, Waltham, MA, USA), and integrity was assessed on an Agilent 2100 Bioanalyzer (Agilent Technologies Inc., Santa Clara, CA, USA); samples with RIN ≥ 8.0 and 28S/18S ratio ≥ 1.8 proceeded to library construction (yield ≥ 500 ng) [46].

Small RNA libraries were generated with the NEBNext^®^ Multiplex Small RNA Library Prep Set for Illumina (v4.1 chemistry; New England Biolabs Inc., Ipswich, MA, USA; Cat. no. E7580) using 3′ and 5′ adapters specific for microRNAs. After reverse transcription and 15 cycles of PCR, the libraries were size-selected (145–160 bp) on 6% Novex TBE-PAGE gels (Thermo Fisher Scientific, Waltham, MA, USA), purified with the Monarch^®^ DNA Gel Extraction Kit (New England Biolabs Inc., Ipswich, MA, USA), and quality-checked on a Bioanalyzer High Sensitivity DNA chip. Twelve uniquely indexed libraries were pooled equimolarly (4 nM each) and sequenced on an Illumina NextSeq 500 platform (Illumina Inc., San Diego, CA, USA) using High-Output v2.5 (single-end, 75 bp), targeting ≥15 million raw reads per sample. All sequencing runs included 5% PhiX spike-in (v3, Illumina Inc., San Diego, CA, USA) for calibration [47].

### 4.7. Bioinformatic Analysis of Small RNA-Seq Data

Sequencing reads were processed with a dedicated small RNA bioinformatics pipeline. First, raw reads underwent quality control using FastQC (v0.11.9, Babraham Bioinformatics, Cambridge, UK) to identify any issues. Adapter sequences introduced by the library prep were then trimmed from reads using Cutadapt (v4.4, Python Package Index, USA), with parameters set to remove 3′ adapters specific to the Illumina small RNA kit and discard reads shorter than 15 nt after trimming [48]. On average, over 95% of reads had adapters successfully removed, and these adapter-trimmed reads were used for alignment. The reads were mapped to the human genome reference (GRCh38) using the Bowtie aligner (v1.3.1), with settings optimized for small RNA (seed length 20, allowing up to 1 mismatch, and suppressing multiple alignments per read) [49]. The alignment was performed with a two-step strategy, as follows: reads were first aligned to a known miRNA reference index derived from miRBase v22 mature miRNA sequences, and any reads that did not align in this step were subsequently aligned to the full genome to detect other small RNAs or novel loci. Aligned reads were then quantified to known miRNAs. For known miRNAs, a read was counted towards a miRNA if it mapped (with zero mismatches) to the mature miRNA sequence or the corresponding hairpin locus in the genome without a better match elsewhere. The read counts for each miRNA were compiled into a count matrix, with an average of ~10 million reads mapped to the miRNA per sample. Read count normalization and differential expression analysis were carried out in R (v4.1.2) using the Bioconductor package DESeq2 (v1.48.2). Raw counts were imported into DESeq2, which uses size factor normalization (median ratio method) to account for library depth differences. The differential expression between experimental groups was assessed using negative binomial generalized linear models, as implemented in DESeq2 [50]. Pairwise comparisons were made to identify the miRNAs regulated by each treatment (e.g., secretome vs. control, QMR^®^ vs. control, combination vs. single treatments, etc.), including interaction effects if any, according to the experimental design. The model included factors for treatment group, time point, and their interaction, and dispersion estimates were moderated to improve accuracy given the small number of replicates. The statistical significance of differential expression was determined using Wald tests, and the resulting *p*-values were adjusted for multiple testing using the Benjamini–Hochberg false discovery rate (FDR) procedure [51]. MiRNAs with an adjusted *p*-value of <0.05 were considered significantly differentially expressed. Additionally, a minimum fold-change threshold of 1.5-fold (|log2FC| ≥ 0.585) was applied to define biologically relevant deregulation. These combined criteria ensured that the identified miRNAs exhibited robust changes in expression. All major data analysis steps were validated by using alternative tools or parameters (for example, mapping was cross-checked with STAR aligner, and a subset of differentially expressed miRNAs was confirmed by qRT-PCR, see Section 4.8 below) to ensure the robustness of the results.

### 4.8. Validation of miRNA Expression by RT-qPCR

To corroborate the small-RNA-seq data, a validation assay based on SYBR^™^ Green real-time PCR was carried out. Total RNA (200 ng per sample) was reverse-transcribed with the miScript II RT kit (QIAGEN), which employs a poly-adenylation step followed by cDNA synthesis with an oligo-dT-universal primer, thereby converting mature miRNAs into amplifiable templates [52]. Quantitative PCR was performed on a QuantStudio^™^ 6 Flex system (Thermo Fisher Scientific) with SYBR^™^ Green PCR Master Mix and miScript Primer Assays specific for three deregulated miRNAs (miR-21-5p, miR-34a-5p, and miR-146a-5p) selected on the basis of statistical significance, FC magnitude, and functional relevance. The cycling conditions comprised an initial denaturation at 95 °C for 10 s followed by 40 cycles of 95 °C for 5 s and 60 °C for 30 s; a melt-curve analysis confirmed the specificity of each amplicon. All reactions were run in technical triplicate, and three independent biological replicates were analyzed per condition. Expression levels were normalized to the small nucleolar RNA RNU6B, and relative abundance was calculated with the 2^−ΔΔCt^ method. The statistical significance between groups was assessed with two-tailed Student’s *t*-tests, accepting *p* < 0.05 as significant [53].

### 4.9. miRNA Target Prediction and Functional Enrichment

To interpret the potential impact of the differentially expressed miRNAs, in silico target gene prediction was performed. The predicted mRNA targets of each significantly deregulated miRNA were identified using multiple databases: TargetScan (Release 8.0) and miRDB were the primary resources [54]. For TargetScan, target genes with context++ score percentile ≤ top 1% (indicating strong predicted binding) were retrieved for each miRNA. From miRDB, targets with a prediction score of ≥80 were selected. The results from these two algorithms were combined, and only genes predicted by at least one of the algorithms (typically yielding hundreds of candidates per miRNA) were retained as potential targets. In cases where multiple miRNAs were significantly altered, an integrated target list was compiled by taking the union of all predicted targets for the upregulated miRNAs and separately for the downregulated miRNAs. To gain insight into the biological processes and pathways affected, we performed functional enrichment analysis on the predicted target gene sets using g:Profiler (version e108_eg53). Enrichment was tested for Gene Ontology (GO) terms (Biological Process, Molecular Function, and Cellular Component), Kyoto Encyclopedia of Genes and Genomes (KEGG) pathways, Reactome pathways, and transcription factor targets, among others. For each gene list (e.g., targets of upregulated miRNAs), g:Profiler’s g:GOSt module was run with the default settings, using the whole human genome as the statistical background. The g:Profiler tool applies a built-in multiple testing correction algorithm (g:SCS), which is thresholded roughly equivalently to an FDR of <0.05 [55]. Only terms with an adjusted *p*-value of <0.05 were deemed significantly enriched. The enrichment results were further filtered to focus on processes relevant to cartilage biology and inflammation, given the experimental context. Redundant terms were condensed by semantic similarity, and the most significant GO terms and pathways are reported. To visualize overlaps, we utilized g:Profiler’s multi-query function to compare the enrichment profiles of target sets from different groups; this analysis highlighted common pathways regulated by multiple miRNAs. Key enriched categories (e.g., extracellular matrix organization, cytokine signaling, and cartilage development) were identified, providing hypotheses regarding the functional consequences of miRNA changes.

### 4.10. miRNA–mRNA Network Construction

To illustrate the regulatory interactions, a network was constructed linking the differentially expressed miRNAs to their predicted target genes, especially those associated with enriched pathways or of particular interest (such as cartilage matrix molecules or inflammation-related genes). We used Cytoscape (v3.9.0) to visualize the miRNA–mRNA interaction network [56]. In the network graph, nodes represent either miRNAs or target genes, and an edge was drawn from a miRNA to a gene if that gene was among the high confidence predicted targets of the miRNA. We further annotated the network with directionality and effect: since miRNAs typically repress gene expression, the edges indicate an inhibitory interaction. The node color was used to denote the experimental regulation direction (miRNA nodes were colored red for upregulated and blue for downregulated; mRNA nodes were shaded according to whether the gene was expected to be up- or downregulated as a consequence). Where available, we integrated gene expression data for target mRNAs from complementary experiments (or public datasets) to support the predicted inverse relationships—for instance, several target genes showed decreased expression in analogous secretome-treated samples, aligning with the upregulation of their cognate miRNAs. The network was analyzed for central hubs and clusters. Notably, a subset of upregulated miRNAs converged on common target genes (forming hub nodes) involved in angiogenesis and extracellular matrix remodeling. Network topology metrics (degree and betweenness centrality) were calculated using the Cytoscape NetworkAnalyzer tool to identify key miRNA regulators [57]. This integrated network approach facilitated a systems-level understanding of how the secretome and QMR^®^ treatments could modulate gene expression programs via miRNAs.

## 5. Conclusions

In summary, we demonstrated that QMR^®^ stimulation, alone and in combination with PDB secretome, modulates the miRNA landscape of oxidatively stressed RPE cells. The treatments elicited distinct miRNA signatures that were predicted to regulate pathways related to antioxidant defense, apoptosis, and inflammation, providing a molecular rationale for exploring their potential regulatory roles, although functional confirmation is still required. Importantly, our data also showed that QMR^®^ alone produces significant effects in this in vitro AMD-mimicking model: it improved cell viability, upregulated anti-inflammatory and antioxidant miRNAs (e.g., miR-146a-5p and miR-590-3p), and suppressed pro-apoptotic signals (e.g., miR-34a-5p and miR-155-5p). These effects are consistent with the modulation of pathways involved in redox homeostasis, inflammation, and survival, such as NF-κB, p53, and FoxO signaling. Notably, QMR^®^ induced a sustained shift in the miRNome persisting for at least 72 h, indicating a durable reprogramming of the RPE post-transcriptional landscape. This long-lasting modulation may underpin the prolonged bioeffects previously reported for QMR^®^. It should be noted, however, that in our experiments, a strong cytoprotective effect was observed with QMR^®^ alone, while secretome and the combined treatment did not provide additional short-term survival benefits. Thus, further studies are required to establish whether the additive effects of secretome treatment may improve this broader reprogramming, translating into functional advantages. Overall, these findings provide mechanistic insights into how QMR^®^ and secretome treatments reprogram RPE miRNA networks under oxidative stress. While they provide exploratory insights into potential non-invasive, cell-free interventions, translation to AMD therapy will require extensive in vivo validation in retinal degeneration models, followed by rigorous clinical assessment of safety, delivery, and efficacy.

## Figures and Tables

**Table 1:** Significantly deregulated miRNAs in RPE cells under oxidative stress (by treatment and time). List of miRNAs with significant differential expression (FDR < 0.05) in retinal pigment epithelial (RPE) cells exposed to oxidative stress, for each treatment condition—QMR^®^, PDB secretome, and combined QMR^®^ + secretome—at 24 h and 72 h. Values shown are log_2_FC relative to untreated stressed control, with corresponding FDR-adjusted *p*-values. Comparisons: OX = TBHP oxidative stress; QMR^®^ = quantum molecular resonance treatment; Secretome = patient blood-derived (PDB) secretome; Control = untreated unstressed control. Each treatment was applied to oxidatively stressed cells except the unstressed control comparisons.

Comparison	Time	miRNA	log_2_FC	FDR
QMR^®^ vs. Control	24 h	hsa-miR-21-5p	1.46	0.003
	24 h	hsa-miR-126-3p	0.85	0.02
	24 h	hsa-miR-146a-5p	1.02	0.015
	24 h	hsa-miR-34a-5p	−1.23	0.009
	24 h	hsa-miR-155-5p	−0.98	0.018
	72 h	hsa-miR-21-5p	2.2	0.0005
	72 h	hsa-miR-146a-5p	1.77	0.001
	72 h	hsa-miR-126-3p	1.05	0.01
	72 h	hsa-miR-29b-3p	1.19	0.03
	72 h	hsa-miR-34a-5p	−1.68	0.0008
	72 h	hsa-miR-155-5p	−1.31	0.004
Secretome vs. Control	24 h	hsa-miR-146a-5p	1.1	0.012
	24 h	hsa-miR-21-5p	0.78	0.027
	24 h	hsa-miR-9-5p	0.95	0.021
	24 h	hsa-miR-126-3p	1.34	0.005
	24 h	hsa-let-7f-5p	−0.81	0.034
	24 h	hsa-miR-34a-5p	−1.1	0.011
	24 h	hsa-miR-155-5p	−1.05	0.016
	72 h	hsa-miR-146a-5p	1.98	0.0008
	72 h	hsa-miR-21-5p	1.53	0.003
	72 h	hsa-miR-204-5p	1.21	0.009
	72 h	hsa-miR-126-3p	0.88	0.025
	72 h	hsa-let-7f-5p	−1.12	0.006
	72 h	hsa-miR-34a-5p	−1.79	0.0004
	72 h	hsa-miR-155-5p	−1.43	0.002
QMR^®^ + Secretome vs. Control	24 h	hsa-miR-146a-5p	2.45	0.0003
	24 h	hsa-miR-21-5p	2.02	0.001
	24 h	hsa-miR-126-3p	1.2	0.008
	24 h	hsa-miR-200a-3p	1.05	0.017
	24 h	hsa-miR-34a-5p	−2.05	0.0001
	24 h	hsa-miR-155-5p	−1.82	0.0009
	24 h	hsa-let-7f-5p	−0.95	0.028
	72 h	hsa-miR-146a-5p	2.1	0.0002
	72 h	hsa-miR-21-5p	1.67	0.0007
	72 h	hsa-miR-126-3p	1.3	0.004
	72 h	hsa-miR-204-5p	1.45	0.002
	72 h	hsa-miR-34a-5p	−2.2	0.0001
	72 h	hsa-miR-155-5p	−1.55	0.001
	72 h	hsa-let-7f-5p	−1.3	0.0005
	72 h	hsa-miR-210-3p	−0.78	0.032
OX vs. Control	24 h	hsa-miR-21-5p	1.5	0.004
	72 h	hsa-miR-34a-5p	2.1	0.001
	72 h	hsa-miR-146a-5p	1.8	0.005
QMR^®^ vs. Control	24 h	hsa-miR-223-3p	1.2	0.02
	72 h	hsa-miR-21-5p	1.3	0.015
	72 h	hsa-miR-146a-5p	1.0	0.03
Secretome vs. Control	24 h	hsa-miR-126-3p	1.1	0.04
	72 h	hsa-miR-21-5p	1.4	0.01
QMR^®^ + Secretome vs. Control	24 h	hsa-miR-21-5p	0.9	0.048
OX vs. Control	24 h	hsa-miR-200a-3p	−0.8	0.018
	72 h	hsa-miR-204-5p	−1.1	0.007
	72 h	hsa-miR-211-5p	−1.3	0.003
QMR^®^ vs. Control	72 h	hsa-miR-204-5p	−0.6	0.04
Secretome vs. Control	72 h	hsa-miR-211-5p	−0.7	0.033
QMR^®^ vs. OX	24 h	hsa-miR-34a-5p	−0.9	0.012
	72 h	hsa-miR-146a-5p	−0.7	0.02
Secretome vs. OX	24 h	hsa-miR-21-5p	−1.0	0.005
	72 h	hsa-miR-34a-5p	−0.8	0.015
QMR^®^ + Secretome vs. OX	24 h	hsa-miR-21-5p	−1.4	0.001
	24 h	hsa-miR-34a-5p	−1.1	0.004
	72 h	hsa-miR-146a-5p	−1.2	0.008

**Table 2:** Top enriched pathways among predicted targets of deregulated miRNAs (by treatment and time). Key enriched biological pathways (Gene Ontology Biological Process, KEGG, and Reactome) identified from predicted target genes of the significantly deregulated miRNAs for each treatment and time point. For each condition, the most significantly enriched Gene Ontology (GO BP) terms, KEGG pathways, and Reactome pathways are listed, with corrected *p*-values (FDR) and the number of target genes involved in each term. Pathway enrichment analysis was performed using g:Profiler on the union of predicted targets (from TargetScan and miRDB) for each set of deregulated miRNAs. All listed terms are significant after multiple-testing correction (adj. *p* < 0.05).

Treatment and Time	Pathway/Term (Category)	Adjusted *p*-Value	Genes (n°)
QMR^®^ 24 h	Inflammatory response (GO BP)	8.0 × 10^−4^	18
	Regulation of apoptotic process (GO BP)	1.2 × 10^−3^	15
	NF-κB signaling pathway (KEGG)	3.0 × 10^−3^	8
	Cytokine signaling in immune system (Reactome)	2.0 × 10^−3^	12
QMR^®^ 72 h	Cellular response to oxidative stress (GO BP)	5.0 × 10^−5^	20
	Regulation of cell cycle (GO BP)	8.0 × 10^−4^	18
	p53 signaling pathway (KEGG)	1.0 × 10^−4^	10
	Cellular senescence and autophagy (Reactome)	1.0 × 10^−3^	9
Secretome 24 h	Angiogenesis (GO BP)	5.0 × 10^−4^	14
	Regulation of cell proliferation (GO BP)	1.0 × 10^−3^	16
	PI3K–Akt signaling pathway (KEGG)	2.0 × 10^−3^	10
	TGF-β receptor signaling (Reactome)	8.0 × 10^−4^	9
Secretome 72 h	Regulation of inflammatory response (GO BP)	4.0 × 10^−4^	17
	Positive regulation of autophagy (GO BP)	5.0 × 10^−3^	8
	FoxO signaling pathway (KEGG)	1.0 × 10^−3^	12
	Extracellular matrix organization (Reactome)	3.0 × 10^−4^	10
QMR^®^ + Secretome 24 h	Regulation of ROS metabolic process (GO BP)	1.0 × 10^−5^	22
	Inflammatory response (GO BP)	2.0 × 10^−4^	20
	TNF signaling pathway (KEGG)	7.0 × 10^−4^	9
	Cellular senescence (Reactome)	4.0 × 10^−4^	10
QMR^®^ + Secretome 72 h	Response to oxidative stress (GO BP)	5.0 × 10^−6^	25
	Regulation of cell proliferation (GO BP)	1.0 × 10^−4^	18
	HIF-1 signaling pathway (KEGG)	3.0 × 10^−4^	11
	Apoptosis (Reactome)	2.0 × 10^−4^	12

**Table 3:** Summary of key molecular effects observed under each treatment condition. Overview of the principal molecular changes induced by QMR^®^ and PDB secretome treatments (alone or combined) in oxidatively stressed RPE cells, highlighting representative deregulated miRNAs, their expected functional consequences, and major affected pathways. Arrows (↑/↓) indicate up- or downregulation of the miRNA. Key pathways listed are derived from enrichment analysis and known targets of the miRNAs: for example, QMR^®^ treatment upregulates miR-146a and downregulates miR-155, consistent with dampening NF-κB inflammatory signaling; secretome elevates miR-204 which targets TGF-β/EMT factors, consistent with reduced fibrosis and senescence, etc. Combined QMR^®^ + secretome results in the broadest restoration of normal cellular homeostasis, targeting multiple stress response and survival pathways.

Condition	Key Deregulated miRNAs	Expected Effect (Functional Outcome)	Major Affected Pathways
QMR^®^	miR-21 ↑, miR-146a ↑, miR-126 ↑; miR-34a ↓, miR-155 ↓	Anti-inflammatory and anti-apoptotic shift, promoting cell survival	NF-κB/TNF inflammatory signaling; p53-mediated apoptosis
Secretome	miR-146a ↑, miR-21 ↑, miR-204 ↑; miR-155 ↓, let-7f ↓, miR-34a ↓	Pro-survival and pro-regenerative response (reduced senescence, enhanced cell viability and proliferation)	PI3K–Akt survival pathway; TGF-β/EMT signaling suppression; inflammatory cytokine signaling
QMR^®^ + Secretome	miR-146a ↑, miR-21 ↑, miR-126 ↑, miR-204 ↑; miR-34a ↓, miR-155 ↓, let-7f ↓	Strongly anti-inflammatory, anti-apoptotic, and anti-senescent effect, restoring a protective homeostatic state	Oxidative stress response pathways (FoxO, HIF-1); cell cycle/senescence (p53, telomere) pathways; NF-κB inflammatory pathway

**Table 4:** Summary of representative dysregulated miRNAs identified in ARPE-19 cells under oxidative stress and their validated/predicted mRNA targets, according to TargetScan and miRDB. Listed targets are associated with stress-relevant pathways including inflammation, apoptosis, oxidative stress, and fibrosis. ↓ decrease of pathway activities, ↑ increase of pathway activities.

miRNA (Direction)	Validated/Predicted Targets	Associated Pathway	Expected Functional Effect
miR-590-3p ↑	*NLRP1*, *NOX4*	Inflammasome, ROS generation	↓ Pyroptosis, ↓ ROS
miR-146a-5p ↑	*IRAK1*, *TRAF6*, *IL-6*, *VEGFA*	NF-κB, Inflammation	↓ Cytokines, ↓ Angiogenesis
miR-34a-5p ↓	*SIRT1*, *BCL2*	Apoptosis, oxidative defense	↑ Survival, ↑ Anti-oxidant
miR-29b-3p ↑	*COL1A1*, *ZEB1/2*	Fibrosis, EMT	↓ ECM deposition
miR-204-5p ↑	*MITF*, *TGF-β* pathway components	Regeneration, anti-fibrosis	↑ Differentiation, ↓ EMT

## Data Availability

The data presented in this study will be made available by the authors on request.

## Supplementary Materials

The following supporting information can be downloaded at: https://www.mdpi.com/article/10.3390/ijms26178614/s1.

## Abbreviations

The following abbreviations are used in this manuscript:

miRNA	microRNA
QMR^®^	Quantum Molecular Resonance
PBD	Patient blood dependent
RPE	Retinal Pigment Epithelium
ROS	Reactive Oxygen Species
tBHP	tert-Butyl hydroperoxide
DEG	Differentially Expressed Gene
FDR	False Discovery Rate
FC/log_2_FC	Fold Change/log base 2-Fold Change
GO	Gene Ontology
KEGG	Kyoto Encyclopedia of Genes and Genomes
EMT	Epithelial-to-Mesenchymal Transition
ECM	Extracellular Matrix
IL	Interleukin
IL1B/IL6R	Interleukin-1 beta/Interleukin-6 receptor
NF-κB	Nuclear Factor kappa-light-chain-enhancer of activated B cells
TNF	Tumor Necrosis Factor
mRNA	Messenger RNA
RT-qPCR	Reverse Transcription quantitative Polymerase Chain Reaction
TS	TargetScan
DB	miRDB
CTRL	Untreated control condition
OX	Oxidative stress condition
Sec	Secretome from PDB
Q + S	Combined QMR^®^ and Secretome treatment
MAPK	Mitogen-Activated Protein Kinase
HIF-1	Hypoxia-Inducible Factor 1
PI3K–Akt	Phosphoinositide 3-kinase–Protein kinase B pathway
TGF-β	Transforming Growth Factor beta
NFE2L2/NRF2	Nuclear factor erythroid 2–related factor 2
NLRP3	NOD-like receptor family pyrin domain-containing 3
CFH	Complement Factor H
STAT1/3	Signal Transducer and Activator of Transcription 1/3
RELA	v-rel avian reticuloendotheliosis viral oncogene homolog A (p65 subunit of NF-κB)
IRAK1	Interleukin-1 Receptor-Associated Kinase 1
TRAF6	TNF Receptor Associated Factor 6
CASP3	Caspase-3
BCL2	B-cell lymphoma 2
TP53	Tumor Protein p53
CDK6	Cyclin-Dependent Kinase 6
SIRT1	Sirtuin 1
ZEB1/2	Zinc finger E-box-binding homeobox 1/2
MET	Mesenchymal–epithelial transition factor (HGFR)
NOX4	NADPH oxidase 4
LC3	Microtubule-associated protein 1A/1B-light chain 3
HMOX1	Heme oxygenase 1
FADD	Fas-Associated Death Domain protein
BCLAF1	Bcl-2-associated transcription factor 1
E2F1/2/3	E2F Transcription Factor 1/2/3
FOXO1/3	Forkhead box protein O1/O3
SOD1	Superoxide Dismutase 1
XBP1	X-box binding protein 1
KEAP1	Kelch-like ECH-associated protein 1

## References

[1] Fuhrmann S., Zou C., Levine E.M. (2014). Retinal pigment epithelium development, plasticity, and tissue homeostasis. Exp. Eye Res..

[2] Lakkaraju A., Umapathy A., Tan L.X., Daniele L., Philp N.J., Boesze-Battaglia K., Williams D.S. (2020). The cell biology of the retinal pigment epithelium. Prog. Retin. Eye Res..

[3] Ferrington D.A., Kapphahn R.J., Leary M.M., Atilano S.R., Terluk M.R., Karunadharma P., Chen G.K., Ratnapriya R., Swaroop A., Montezuma S.R. (2016). Increased retinal mtDNA damage in the CFH variant associated with age-related macular degeneration. Exp. Eye Res..

[4] Golestaneh N., Chu Y., Xiao Y.Y., Stoleru G.L., Theos A.C. (2017). Dysfunctional autophagy in RPE, a contributing factor in age-related macular degeneration. Cell Death Dis..

[5] Huang J.J., Xia J., Huang L.L., Li Y.C. (2019). HIF-1alpha promotes NLRP3 inflammasome activation in bleomycin-induced acute lung injury. Mol. Med. Rep..

[6] Pfau M., von der Emde L., de Sisternes L., Hallak J.A., Leng T., Schmitz-Valckenberg S., Holz F.G., Fleckenstein M., Rubin D.L. (2020). Progression of Photoreceptor Degeneration in Geographic Atrophy Secondary to Age-related Macular Degeneration. JAMA Ophthalmol..

[7] Yang Y., Lin Y., Han Z., Wang B., Zheng W., Wei L. (2024). Ferroptosis: A novel mechanism of cell death in ophthalmic conditions. Front. Immunol..

[8] Yu L., Liu P. (2024). cGAS/STING signalling pathway in senescence and oncogenesis. Semin. Cancer Biol..

[9] Zhou M., Geathers J.S., Grillo S.L., Weber S.R., Wang W., Zhao Y., Sundstrom J.M. (2020). Role of Epithelial-Mesenchymal Transition in Retinal Pigment Epithelium Dysfunction. Front. Cell Dev. Biol..

[10] Jung W.K., Park S.B., Yu H.Y., Kim Y.H., Kim J. (2022). Effect of Esculetin on Tert-Butyl Hydroperoxide-Induced Oxidative Injury in Retinal Pigment Epithelial Cells In Vitro. Molecules.

[11] Rabin D.M., Rabin R.L., Blenkinsop T.A., Temple S., Stern J.H. (2013). Chronic oxidative stress upregulates Drusen-related protein expression in adult human RPE stem cell-derived RPE cells: A novel culture model for dry AMD. Aging.

[12] Hanus J., Zhang H., Wang Z., Liu Q., Zhou Q., Wang S. (2013). Induction of necrotic cell death by oxidative stress in retinal pigment epithelial cells. Cell Death Dis..

[13] Diaz-Riascos Z.V., Ginesta M.M., Fabregat J., Serrano T., Busquets J., Buscail L., Cordelier P., Capella G. (2019). Expression and Role of MicroRNAs from the miR-200 Family in the Tumor Formation and Metastatic Propensity of Pancreatic Cancer. Mol. Ther. Nucleic Acids.

[14] Hao Y., Zhou Q., Ma J., Zhao Y., Wang S. (2016). miR-146a is upregulated during retinal pigment epithelium (RPE)/choroid aging in mice and represses IL-6 and VEGF-A expression in RPE cells. J. Clin. Exp. Ophthalmol..

[15] Urbanska K., Stepien P.W., Nowakowska K.N., Stefaniak M., Osial N., Choragiewicz T., Toro M.D., Nowomiejska K., Rejdak R. (2022). The Role of Dysregulated miRNAs in the Pathogenesis, Diagnosis and Treatment of Age-Related Macular Degeneration. Int. J. Mol. Sci..

[16] Usui-Ouchi A., Ouchi Y., Kiyokawa M., Sakuma T., Ito R., Ebihara N. (2016). Upregulation of Mir-21 Levels in the Vitreous Humor Is Associated with Development of Proliferative Vitreoretinal Disease. PLoS ONE.

[17] Trivli A., Karmiris E., Dalianis G., Ruggeri A., Terzidou C. (2023). Evaluating the efficacy of Quantum Molecular Resonance (QMR®) electrotherapy in mixed-type dry eye patients. J. Optom..

[18] Ballesteros-Sanchez A., Sanchez-Gonzalez J.M., Tedesco G.R., Rocha-De-Lossada C., Russo F., Spinelli A., Ingrande I., Borroni D. (2024). Efficacy and Safety of Quantum Molecular Resonance Electrotherapy in Patients with Aqueous-Deficient, Evaporative and Mixed-Type Dry Eye: A Randomized Interventional Study. Ophthalmol. Ther..

[19] Trigo C.M., Rodrigues J.S., Camoes S.P., Sola S., Miranda J.P. (2025). Mesenchymal stem cell secretome for regenerative medicine: Where do we stand?. J. Adv. Res..

[20] Gu C., Draga D., Zhou C., Su T., Zou C., Gu Q., Lahm T., Zheng Z., Qiu Q. (2019). miR-590-3p Inhibits Pyroptosis in Diabetic Retinopathy by Targeting NLRP1 and Inactivating the NOX4 Signaling Pathway. Investig. Ophthalmol. Vis. Sci..

[21] Sabbatinelli J., Giuliani A., Matacchione G., Latini S., Laprovitera N., Pomponio G., Ferrarini A., Svegliati Baroni S., Pavani M., Moretti M. (2021). Decreased serum levels of the inflammaging marker miR-146a are associated with clinical non-response to tocilizumab in COVID-19 patients. Mech. Ageing Dev..

[22] Paolucci T., Pino V., Elsallabi O., Gallorini M., Pozzato G., Pozzato A., Lanuti P., Reis V.M., Pesce M., Pantalone A. (2023). Quantum Molecular Resonance Inhibits NLRP3 Inflammasome/Nitrosative Stress and Promotes M1 to M2 Macrophage Polarization: Potential Therapeutic Effect in Osteoarthritis Model In Vitro. Antioxidants.

[23] Fraccalvieri M., Salomone M., Di Santo C., Ruka E., Morozzo U., Bruschi S. (2017). Quantum molecular resonance technology in hard-to-heal extremity wounds: Histological and clinical results. Int. Wound J..

[24] Daneshmandi L., Shah S., Jafari T., Bhattacharjee M., Momah D., Saveh-Shemshaki N., Lo K.W., Laurencin C.T. (2020). Emergence of the Stem Cell Secretome in Regenerative Engineering. Trends Biotechnol..

[25] Liao Z., Zheng R., Shao G. (2023). Mechanisms and application strategies of miRNA-146a regulating inflammation and fibrosis at molecular and cellular levels (Review). Int. J. Mol. Med..

[26] Mesquida M., Leszczynska A., Llorenc V., Adan A. (2014). Interleukin-6 blockade in ocular inflammatory diseases. Clin. Exp. Immunol..

[27] Serrander L., Cartier L., Bedard K., Banfi B., Lardy B., Plastre O., Sienkiewicz A., Forro L., Schlegel W., Krause K.H. (2007). NOX4 activity is determined by mRNA levels and reveals a unique pattern of ROS generation. Biochem. J..

[28] Ahmad A., Ahsan H. (2020). Biomarkers of inflammation and oxidative stress in ophthalmic disorders. J. Immunoass. Immunochem..

[29] Ochoa Hernandez M.E., Lewis-Lujan L.M., Burboa Zazueta M.G., Del Castillo Castro T., De La Re Vega E., Galvez-Ruiz J.C., Trujillo-Lopez S., Lopez Torres M.A., Iloki-Assanga S.B. (2025). Role of Oxidative Stress and Inflammation in Age Related Macular Degeneration: Insights into the Retinal Pigment Epithelium (RPE). Int. J. Mol. Sci..

[30] Ambati J., Fowler B.J. (2012). Mechanisms of age-related macular degeneration. Neuron.

[31] Marchesi N., Capierri M., Pascale A., Barbieri A. (2024). Different Therapeutic Approaches for Dry and Wet AMD. Int. J. Mol. Sci..

[32] Cruz-Aguilar M., Groman-Lupa S., Jimenez-Martinez M.C. (2023). MicroRNAs as potential biomarkers and therapeutic targets in age-related macular degeneration. Front. Ophthalmol..

[33] Brillante S., Volpe M., Indrieri A. (2024). Advances in MicroRNA Therapeutics: From Preclinical to Clinical Studies. Hum. Gene Ther..

[34] Schnichels S., Paquet-Durand F., Loscher M., Tsai T., Hurst J., Joachim S.C., Klettner A. (2021). Retina in a dish: Cell cultures, retinal explants and animal models for common diseases of the retina. Prog. Retin. Eye Res..

[35] Bonilha V.L. (2014). Retinal pigment epithelium (RPE) cytoskeleton in vivo and in vitro. Exp. Eye Res..

[36] Romano G.L., Platania C.B.M., Drago F., Salomone S., Ragusa M., Barbagallo C., Di Pietro C., Purrello M., Reibaldi M., Avitabile T. (2017). Retinal and Circulating miRNAs in Age-Related Macular Degeneration: An In vivo Animal and Human Study. Front. Pharmacol..

[37] Ahmado A., Carr A.J., Vugler A.A., Semo M., Gias C., Lawrence J.M., Chen L.L., Chen F.K., Turowski P., da Cruz L. (2011). Induction of differentiation by pyruvate and DMEM in the human retinal pigment epithelium cell line ARPE-19. Investig. Ophthalmol. Vis. Sci..

[38] Dhurat R., Sukesh M. (2014). Principles and Methods of Preparation of Platelet-Rich Plasma: A Review and Author’s Perspective. J. Cutan. Aesthet. Surg..

[39] Harrison T.E., Bowler J., Cheng C.I., Reeves K.D. (2023). Optimizing Platelet-Rich Plasma: Spin Time and Sample Source. Bioengineering.

[40] Patel H., Pundkar A., Shrivastava S., Chandanwale R., Jaiswal A.M. (2023). A Comprehensive Review on Platelet-Rich Plasma Activation: A Key Player in Accelerating Skin Wound Healing. Cureus.

[41] Cavallo C., Roffi A., Grigolo B., Mariani E., Pratelli L., Merli G., Kon E., Marcacci M., Filardo G. (2016). Platelet-Rich Plasma: The Choice of Activation Method Affects the Release of Bioactive Molecules. BioMed Res. Int..

[42] Prado-Yupanqui J.W., Ramirez-Orrego L., Cortez D., Vera-Ponce V.J., Chenet S.M., Tejedo J.R., Tapia-Limonchi R. (2025). The Hidden Power of the Secretome: Therapeutic Potential on Wound Healing and Cell-Free Regenerative Medicine-A Systematic Review. Int. J. Mol. Sci..

[43] Sonmez O., Sonmez M. (2017). Role of platelets in immune system and inflammation. Porto Biomed. J..

[44] Afsar E., Kirimlioglu E., Ceker T., Yilmaz C., Demir N., Aslan M. (2020). Effect of ER stress on sphingolipid levels and apoptotic pathways in retinal pigment epithelial cells. Redox Biol..

[45] Thiese M.S., Ronna B., Ott U. (2016). P value interpretations and considerations. J. Thorac. Dis..

[46] Sriram H., Khanka T., Kedia S., Tyagi P., Ghogale S., Deshpande N., Chatterjee G., Rajpal S., Patkar N.V., Subramanian P.G. (2021). Improved protocol for plasma microRNA extraction and comparison of commercial kits. Biochem. Med..

[47] Shore S., Henderson J.M., Lebedev A., Salcedo M.P., Zon G., McCaffrey A.P., Paul N., Hogrefe R.I. (2016). Small RNA Library Preparation Method for Next-Generation Sequencing Using Chemical Modifications to Prevent Adapter Dimer Formation. PLoS ONE.

[48] Potla P., Ali S.A., Kapoor M. (2021). A bioinformatics approach to microRNA-sequencing analysis. Osteoarthr. Cartil. Open.

[49] Langmead B., Trapnell C., Pop M., Salzberg S.L. (2009). Ultrafast and memory-efficient alignment of short DNA sequences to the human genome. Genome Biol..

[50] Love M.I., Anders S., Kim V., Huber W. (2015). RNA-Seq workflow: Gene-level exploratory analysis and differential expression. F1000Research.

[51] Chen Z., Liu J., Ng H.K., Nadarajah S., Kaufman H.L., Yang J.Y., Deng Y. (2011). Statistical methods on detecting differentially expressed genes for RNA-seq data. BMC Syst. Biol..

[52] Sharbati-Tehrani S., Kutz-Lohroff B., Bergbauer R., Scholven J., Einspanier R. (2008). miR-Q: A novel quantitative RT-PCR approach for the expression profiling of small RNA molecules such as miRNAs in a complex sample. BMC Mol. Biol..

[53] Pfaffl M.W. (2001). A new mathematical model for relative quantification in real-time RT-PCR. Nucleic Acids Res..

[54] Dias B.D.S., Diniz L.F.A., Correa L.D., Souza R.P., Ferreira L.T., Pasqualin D.D.C., Cicco R., Silva E., Severino P. (2025). Comparative analysis of miRNA-mRNA interaction prediction tools based on experimental head and neck cancer data. Einstein.

[55] Reimand J., Isserlin R., Voisin V., Kucera M., Tannus-Lopes C., Rostamianfar A., Wadi L., Meyer M., Wong J., Xu C. (2019). Pathway enrichment analysis and visualization of omics data using g:Profiler, GSEA, Cytoscape and EnrichmentMap. Nat. Protoc..

[56] Leon L.E., Calligaris S.D. (2017). Visualization and Analysis of MiRNA-Targets Interactions Networks. Methods Mol. Biol..

[57] Doncheva N.T., Assenov Y., Domingues F.S., Albrecht M. (2012). Topological analysis and interactive visualization of biological networks and protein structures. Nat. Protoc..

