# Supplementary material for: QMR® and Patient Blood-Derived Secretome Modulate RPE microRNA Networks Under Oxidative Stress
Source: Int J Mol Sci. 2025 Sep 4;26(17):8614. doi: 10.3390/ijms26178614 (PMC12429622; doi:10.3390/ijms26178614)
Supplement: Supplementary file 1 [file ijms-26-08614-s001.zip › ijms-3826605-supplementary.pdf]

Supplementary Table S1. *Differential expression of all detected miRNAs at 24 h (oxidative stress and treatments).* Listed are log_2FC and FDR-adjusted p-values for each miRNA, in each 24 h comparison: oxidative stress vs. control (OX vs CTRL), QMR® treatment vs. untreated oxidatively stressed (QMR vs OX), MSC secretome vs. OX (Sec vs OX), and combined QMR®+Secretome vs. OX (Q+S vs OX). Positive log_2FC indicates higher miRNA expression in the first condition of the comparison, negative indicates lower. Significant changes (FDR < 0.05) are in **bold**.

miRNA	log_2FC (OX vs CTRL 24 h)	FDR (OX vs CTRL)	log_2FC (QMR® vs OX 24 h)	FDR (QMR® vs OX)	log_2FC (Sec vs OX 24 h)	FDR (Sec vs OX)	log_2FC (Q+S vs OX 24 h)	FDR (Q+S vs OX)
**miR-21-5p**	+0.80	0.010	**+1.46**	**0.003**	**+0.78**	**0.027**	**+2.02**	**0.001**
**miR-34a-5p**	**+1.10**	**0.0005**	**–1.23**	**0.009**	**–1.10**	**0.011**	**–2.05**	**0.0001**
**miR-146a-5p**	+0.20	0.150	**+1.02**	**0.015**	**+1.10**	**0.012**	**+2.45**	**0.0003**
**miR-155-5p**	+0.10	0.340	**–0.98**	**0.018**	**–1.05**	**0.016**	**–1.82**	**0.0009**
**miR-126-3p**	–0.05	0.880	**+0.85**	**0.020**	**+1.34**	**0.005**	**+1.20**	**0.008**
**miR-29b-3p**	–0.20	0.220	+0.10	0.760	+0.05	0.910	**–0.70**	**0.040**
**miR-9-5p**	+0.05	0.940	–0.10	0.800	**+0.95**	**0.021**	+0.20	0.500
**let-7f-5p**	–0.15	0.480	–0.20	0.390	**–0.81**	**0.034**	**–0.95**	**0.028**
**miR-200a-3p**	–0.05	0.870	–0.10	0.650	+0.10	0.570	**+1.05**	**0.017**
**miR-200c-3p**	–0.08	0.820	–0.12	0.730	–0.05	0.900	**+0.90**	**0.045**
**miR-204-5p**	**–1.00**	**0.004**	+0.10	0.800	+0.30	0.310	+0.20	0.350
**miR-211-5p**	**–0.95**	**0.005**	+0.02	0.970	+0.05	0.920	–0.10	0.780
**miR-210-3p**	+0.40	0.110	+0.25	0.220	+0.10	0.600	–0.20	0.320
**miR-27a-3p**	+0.05	0.930	–0.60	0.050	–0.20	0.500	**–1.50**	**0.002**
**miR-590-3p**	+0.10	0.800	**+1.00**	**0.010**	–0.20	0.400	+0.15	0.550
**miR-214-3p**	–0.10	0.770	+0.05	0.920	–0.08	0.880	**+1.00**	**0.008**
(other miRNAs)	*(no significant change)*		*(no significant change)*		*(no significant change)*		*(no significant change)*	

Supplementary Table S2. *Differential expression of all detected miRNAs at 72 h.* Format and comparisons as in S1A, for 72 h post-stress. CTRL = untreated control; OX = oxidative stress (tBHP) control; QMR® = Quantum Molecular Resonance treatment; Sec = MSC secretome treatment; Q+S = combined treatment.

miRNA	log_2FC (OX vs CTRL 72 h)	FDR (OX vs CTRL)	log_2FC (QMR® vs OX 72 h)	FDR (QMR® vs OX)	log_2FC (Sec vs OX 72 h)	FDR (Sec vs OX)	log_2FC (Q+S vs OX 72 h)	FDR (Q+S vs OX)
**miR-21-5p**	**+1.80**	**0.0002**	+0.20	0.400	**+1.53**	**0.003**	**+1.67**	**0.0007**
**miR-34a-5p**	**+1.50**	**0.0008**	–0.20	0.360	–0.15	0.480	**–2.20**	**0.0001**
**miR-146a-5p**	**+2.10**	**0.0003**	–0.10	0.780	+0.50	0.090	**+2.10**	**0.0002**
**miR-155-5p**	+0.30	0.240	+0.05	0.920	–0.20	0.500	**–1.55**	**0.001**
**miR-126-3p**	–0.10	0.670	**+1.05**	**0.010**	**+0.88**	**0.025**	**+1.30**	**0.004**
**miR-29b-3p**	–0.20	0.310	**+1.19**	**0.030**	–0.10	0.820	+0.05	0.900
**miR-9-5p**	+0.10	0.840	+0.02	0.990	–0.05	0.950	+0.15	0.670
**let-7f-5p**	+0.05	0.930	+0.10	0.800	–0.30	0.120	**–1.12**	**0.006**
**miR-200a-3p**	–0.50	0.060	+0.20	0.330	+0.10	0.710	**+0.80**	**0.010**
**miR-200c-3p**	–0.30	0.210	+0.10	0.600	+0.05	0.880	+0.20	0.340
**miR-204-5p**	**–1.20**	**0.009**	+0.08	0.850	**+1.21**	**0.009**	**+1.45**	**0.002**
**miR-211-5p**	**–1.30**	**0.010**	–0.10	0.780	+0.05	0.920	–0.20	0.400
**miR-210-3p**	+0.60	0.055	+0.30	0.130	+0.10	0.700	**–0.78**	**0.032**
**miR-27a-3p**	+0.15	0.600	–0.50	0.070	–0.20	0.480	**–1.50**	**0.0008**
**miR-590-3p**	+0.10	0.800	–0.20	0.400	**+0.70**	**0.015**	**+2.00**	**0.0005**
**miR-214-3p**	–0.05	0.950	–0.10	0.730	+0.05	0.900	**+1.00**	**0.005**
(other miRNAs)	*(no significant change)*		*(no significant change)*		*(no significant change)*		*(no significant change)*	

Supplementary Table S3. *Predicted target genes of significantly deregulated miRNAs.* For each miRNA that showed significant up- or down-regulation in at least one condition (from Supplementary Table S1), all predicted gene targets are listed. Target predictions were obtained by combining the results from TargetScan (human v7.2) and miRDB (v6.0) databases. The union of predicted targets from both databases is shown for each miRNA (duplicates removed). **Bolded** gene names denote targets that are reported by *both* algorithms (high-confidence predictions). *Italicized* genes are examples with well-established roles in pathways relevant to RPE oxidative stress (literature-supported targets). *(Note: This is an extensive list; only unique gene symbols are listed per miRNA.)*

miRNA	Targets
**let-7f-5p**	HMGA2, **NRAS**, IGF1R, IL6, E2F2, KRAS, MYC, CDK6, MAP2K4
**miR-9-5p**	**NFKB1**, SIRT1, FOXO1, CXCL8 (IL-8), ONECUT2, JAK1, NOX4, NLRP3, VEGFA
**miR-21-5p**	**PTEN**, **PDCD4**, SPRY1, RECK, BTG2, TPM1, TIMP3, TGFBR3, IL1β, SOCS5
**miR-27a-3p**	*NFE2L2 (NRF2)*, **GSK3B**, SFRP1, KEAP1, CCL2, TSC1, APOBEC3G, PPARG
**miR-29b-3p**	COL1A1, DNMT3B, MCL1, SPARC, ELN, PDGF-C, ECM2, THBS1, HDAC4, **TP53**
**miR-34a-5p**	**SIRT1**, **BCL2**, CCND1, CDK6, MET, CASP3, NOTCH1, HMOX1, CYCS, MYCN
**miR-126-3p**	PIK3R2, SPRED1, VCAM1, IRS1, CRK, EGFL7, CX3CL1, HOXA9, **VEGFA**, ADAM9
**miR-146a-5p**	**IRAK1**, **TRAF6**, IL6R, RELA, SMAD4, CFH, NOD1, BCL2A1, CARD10, STAT1
**miR-155-5p**	**SOCS1**, INPP5D (*SHIP1*), CFH, FOXO3, TP53INP1, RIPK1, CASP2, IL1B, FADD
**miR-200a-3p**	**ZEB1**, **ZEB2**, TGFBR2, SMAD3, EP300, β-Catenin (CTNNB1), IL8, FN1, MITF
**miR-200c-3p**	ZEB1, ZEB2, ETS1, FGF7, **BMI1**, JUN, LMNA, TCF4, **EPHA2**, CHEK1
**miR-204-5p**	BCL2, MAP1LC3B, MEIS2, AXIN2, CREB1, ATF2, EDEM1, SNAI1, FZD4, LC3
**miR-210-3p**	ISCU, HIF3A, E2F3, ACVR1B, NDUFA4, EFNA3, DRD2, HGFR (MET), CASP8AP2
**miR-211-5p**	*RASGRF1*, *TRPM1*, *MITF*, TGFBR1, JAK2, IGF1R, NFAT5, **STAT3**, SOX11
**miR-214-3p**	EZH2, *ATF4*, ITGA5, β-Catenin (CTNNB1), ACTR2, KPNA3, SNCA, SOD1, **XBP1**
**miR-590-3p**	*NOX4*, *NLRP1*, CHD2, PRRX1, HMOX1, E2F1, BCLAF1, TXNIP, KEAP1, CCL5

(Full target gene lists were used for pathway analysis. High-confidence/common targets are bold; selected known stress-response targets are italicized for reference.)

Supplementary Table S4. *Comprehensive pathway enrichment analysis for miRNA target gene sets.* Complete Gene Ontology Biological Process (GO BP), KEGG pathway, and Reactome pathway enrichment results (FDR < 0.05) for predicted targets of deregulated miRNAs in each condition. Each section corresponds to one comparison (treatment vs control, at a given time) and lists all significantly enriched terms identified by g:Profiler for that target gene set. Terms are sorted within each category by adjusted p-value. (The top pathways highlighted in the main text/Table 2 are included here among the full results.)

Condition	Category	Term	FDR	Genes
**OX vs CTRL (72h)**	GO	Response to oxidative stress	2.3e-06	24
	GO	Intrinsic apoptotic signaling pathway	4.1e-05	18
	KEGG	FoxO signaling pathway	0.00032	10
	KEGG	p53 signaling pathway	0.0005	8
	Reactome	Detoxification of Reactive Oxygen Species	8.5e-05	6
	Reactome	Oxidative stress-induced senescence	0.00011	5
**QMR® 24h**	GO	Inflammatory response	0.0008	18
	GO	Regulation of apoptotic process	0.0012	15
	GO	Response to oxidative stress	0.005	10
	KEGG	NF-Œ∫B signaling pathway	0.003	8
	KEGG	TNF signaling pathway	0.01	6
	Reactome	Cytokine signaling in immune system	0.002	12
	Reactome	Apoptosis	0.015	9
**QMR® 72h**	GO	Cellular response to oxidative stress	5,00E-05	20
	GO	Regulation of cell cycle	0.0008	18
	GO	Cellular senescence	0.004	12
	KEGG	p53 signaling pathway	0.0001	10
	KEGG	Apoptosis	0.007	7
	Reactome	Senescence and autophagy	0.001	9
	Reactome	DNA damage response	0.01	5
**Secretome 24h**	GO	Angiogenesis	0.0005	14
	GO	Regulation of cell proliferation	0.001	16
	GO	Regulation of inflammatory response	0.0035	11
	GO	Cell migration	0.01	8
	KEGG	PI3K,Akt signaling pathway	0.002	10
	KEGG	TGF-beta signaling pathway	0.008	6
	Reactome	TGF-beta receptor signaling	0.0008	9
	Reactome	Extracellular matrix organization	0.005	7
**Secretome 72h**	GO	Regulation of inflammatory response	0.0004	17
	GO	Positive regulation of autophagy	0.005	8
	GO	Response to oxidative stress	0.01	7
	KEGG	FoxO signaling pathway	0.001	12
	KEGG	MAPK signaling pathway	0.009	7
	Reactome	Extracellular matrix organization	0.0003	10
	Reactome	Signal transduction	0.02	15
**QMR® + Secretome 24h**	GO	Regulation of reactive oxygen species metabolic process	1,00E-05	22

	GO	Inflammatory response	0.0002	20
	GO	Regulation of apoptotic process	0.0009	15
	GO	Immune system process	0.005	25
	KEGG	TNF signaling pathway	0.0007	9
	KEGG	NF-ŒʃB signaling pathway	0.002	8
	Reactome	Cellular senescence	0.0004	10
	Reactome	Cytokine signaling in immune system	0.001	13
	Reactome	Autophagy	0.01	6
	GO	Response to oxidative stress	5,00E-06	25
	GO	Regulation of cell proliferation	0.0001	18
	GO	DNA repair	0.002	8
	GO	Cellular response to stress	0.005	14
**QMR® + Secretome 72h**	KEGG	HIF-1 signaling pathway	0.0003	11
	KEGG	NF-kb signaling pathway	0.0015	10
	Reactome	Apoptosis	0.0002	12
	Reactome	Interleukin-6 signaling	0.003	7
	Reactome	Signal transduction	0.02	16

Supplementary Table S5. *miRNA–mRNA interaction network under oxidative stress (OX vs Control).* Example predicted target interactions for miRNAs significantly altered by oxidative stress, showing inverse changes in target mRNA expression. Up-regulated miRNAs in OX (relative to control) are paired with down-regulated target genes; down-regulated miRNAs (lower in OX) are paired with up-regulated targets. All listed target genes were significantly differentially expressed in OX vs CTRL (FDR < 0.05). "Prediction Source" indicates the database(s) that identify the miRNA–target pairing: TargetScan (TS) and/or miRDB (DB). *These interactions suggest that OX-induced miRNAs (e.g., miR-21, miR-34a, miR-146a) may repress specific mRNAs, whereas OX-suppressed miRNAs (miR-204, miR-211, miR-200a) lead to de-repression of their targets under stress.*

Up-regulated OX miRNAs (higher in OX vs Ctrl) and their down-regulated target genes

miRNA (up in OX)	Predicted Target Gene (down in OX)	Prediction Source	Target gene log_2FC (OX vs Ctrl)	Target FDR (OX vs Ctrl)
**miR-21-5p**	**PDCD4** *(Programmed cell death 4)*	TS & DB	–1.5	0.0005
**miR-34a-5p**	**SIRT1** *(Sirtuin 1)*	TS & DB	–1.0	0.002
**miR-146a-5p**	**IRAK1** *(IL-1 receptor–associated kinase 1)*	TS & DB	–0.7	0.010

Down-regulated OX miRNAs (lower in OX vs Ctrl) and their up-regulated target genes

miRNA (down in OX)	Predicted Target Gene (up in OX)	Prediction Source	Target gene log_2FC (OX vs Ctrl)	Target FDR (OX vs Ctrl)
**miR-204-5p**	**BCL2** *(B-cell lymphoma 2)*	TS & DB	+0.8	0.050
**miR-211-5p**	**IL6R** *(interleukin-6 receptor)*	TS & DB	+0.7	0.020
**miR-200a-3p**	**ZEB1** *(zinc finger E-box-binding homeobox 1)*	TS & DB	+1.2	0.010
